# Unraveling the metabolic network and key bioactive compound accumulation during critical developmental stages of *Gastrodia elata*

**DOI:** 10.3389/fpls.2026.1804747

**Published:** 2026-05-01

**Authors:** Jingling Duan, Yi Tu, Haiyan Zhu, Jiahong Dong, Lei Zhang, Pengzhang Ji

**Affiliations:** 1 School of Chinese Materia Medica & Yunnan Key Laboratory of Sustainable Utilization of Southern Medicine, Yunnan University of Chinese Medicine, Kunming, China; 2 Institute of Medicinal Plant Cultivation, Academy of Southern Medicine, Yunnan University of Chinese Medicine, Kunming, China

**Keywords:** bioactive compound, developmental stages, *Gastrodia elata*, metabolomics analysis, regulatory network

## Abstract

*Gastrodia elata* Blume, a traditional medicinal plant, the dynamic metabolic changes and the mechanisms underlying the accumulation of medicinal components remain poorly understood during its key developmental stages (tubers before bolting, tubers after bolting, stems). This study utilized untargeted metabolomics to systematically analyze the metabolic composition and differences in tubers before bolting, tubers after bolting, and stems of *G. elata* f. *elata*, *G. elata* f. *glauca*, and dark red *G. elata*. We identified a total of 6209 metabolites, encompassing 17 major categories, and of these, 1624 differential metabolites (VIP > 1.5, *P* < 0.05) exhibited significant variations across developmental stages. Lipids, organic acids, and carbohydrates showed marked accumulation in tubers after bolting, while stems exhibited predominant enrichment of flavonoids, coumarins, and lignans. Lipids and flavonoids emerged as key differential metabolites governing the development of *G. elata*. Gastrodin and parishin C, two key bioactive constituents, were found to be significantly increased in both post-bolting tubers and stems (*P* < 0.05). Flavonoid biosynthesis, zeatin metabolism, and phytohormone signal transduction serve as the core metabolic pathways regulating the key developmental stages of *Gastrodia elata*. Meanwhile, amino sugar and nucleotide sugar metabolism, starch and sucrose metabolism, glycolysis/gluconeogenesis, and pentose and glucuronate interconversions form the central energy metabolic network underlying these critical developmental processes in *G. elata*. In addition, 22, 18, and 3 metabolites unique to *G. elata* f. *elata*, *G. elata* f. *glauca*, and dark red *G. elata*, respectively, were identified, which hold promise as potential markers for cultivar discrimination. This study reveals for the first time the molecular regulatory network of *Gastrodia elata* at key developmental stages from a metabolic perspective, providing important theoretical foundations for its growth regulation, quality improvement, and rational resource utilization.

## 1 Introduction

First recorded in Shennong’s Classic of Materia Medica under the name “Chijian” (Ding et al., 2025), *Gastrodia elata* (Tianma) was regarded as a superior-grade medicinal herb with the efficacy of “enhancing qi and strength upon prolonged administration” (Shen et al., 2023). Five varieties are recognized within this species, specifically *G. elata* f. *elata*, *G. elata* f. *viridis*, *G. elata* f. *glauca*, *G. elata* f. *alba*, and *G. elata* f. *flavid* (Wang et al., 2023). *Gastrodia elata* contains various active components, among which the currently isolated and identified ones include phenols and their glycosides, organic acids and their esters, polysaccharides, and sterols (Fan et al., 2021). Phenolic compounds are the main active components of *Gastrodia elata* and can be categorized based on their structures into phenolic glycosides, phenolic aldehydes, phenolic alcohols, and other phenolic derivatives (Du et al., 2025). Gastrodin, a phenolic glycoside, is the primary bioactive constituent and a quality control marker for *G. elata* (Li et al., 2019). P-Hydroxybenzyl alcohol, the aglycone of gastrodin, together with gastrodin, forms the pharmacological basis of its therapeutic effects (Cheng and Deng, 2021). Parishins, isolated from *G. elata*, are phenolic ester glucosides structurally characterized by citric acid derivatives esterified with p-Hydroxybenzyl alcohol or its derivatives; they serve as biotransformation precursors of gastrodin (Kim et al., 2022; Du et al., 2025). Furthermore, *G. elata* contains other phenolic constituents, including phenolic acids (e.g.,p-hydroxybenzaldehyde, vanillin), flavanols (e.g., catechin), and phenylpropanoids (e.g., caffeic acid) (Li et al., 2007; Wang et al., 2007; Zhan et al., 2016). Modern pharmacological studies have demonstrated that *Gastrodia elata* exhibits multi-level neuropharmacological activities. At the cellular and molecular level, gastrodin has been shown to alleviate oxidative stress injury and inhibit neuronal apoptosis by activating the Nrf2/HO-1 signaling pathway (Liu et al., 2018). In various animal models of neurological diseases, *Gastrodia elata* extracts and their active components have demonstrated protective effects against epilepsy, Parkinson’s disease, and cerebral ischemia-reperfusion injury (Wang et al., 2025). Preliminary clinical studies suggest that gastrodin adjuvant therapy may improve symptoms in patients with epilepsy and Parkinson’s disease with mild cognitive impairment; however, high-quality clinical studies remain limited and require further validation (Xiao et al., 2023). In 2019, the National Health Commission and the State Administration for Market Regulation jointly issued a document to include *Gastrodia elata* in the list of medicinal and edible substances (Guo et al., 2022). Xiaocaoba Town, Yiliang County, Zhaotong City, Yunnan Province is the world’s original production area of Tianma. Its annual Tianma output accounts for 97% of Yiliang County’s total production and approximately 10% of the national output, making it truly worthy of the title “Hometown of Tianma” (Chen, 2024). Currently, *Gastrodia elata* (Tianma) cultivation in China has formed three relatively concentrated main production areas: the Qinba Mountain production area centered around Hanzhong in Shaanxi, the Wumeng Mountain production area centered around Zhaotong in Yunnan and Bijie in Guizhou, and the Dabie Mountain production area primarily in Yuexi, Anhui (Ding et al., 2025).

The medicinal quality of *Gastrodia elata* is closely related to its developmental stage. As the medicinal organ, the quality formation of the tuber is tightly associated with the developmental process. Studies have shown that the mature tuber (Jianma) represents the optimal harvest period, during which the accumulation of gastrodin and parishin-type components tends to stabilize (Zhu et al., 2024). However, if not harvested in time and bolting occurs, the tubers often become hollow and lignified, and the contents of bioactive components such as gastrodin and parishins decrease significantly, resulting in loss of medicinal value (Zhang et al., 2011; Shi et al., 2026). It is noteworthy that bolting-induced quality deterioration is not unique to *G. elata*. Early bolting in the medicinal plant *Angelica sinensis* severely affects yield and quality, with bolting rates reaching 30%-50% (Li et al., 2022); similarly, bolting in radish leads to hollowing and cracking of the fleshy roots, severely compromising commercial value (Jin, 2024).

The five primary developmental stages of *Gastrodia elata* are seed, protocorm, juvenile tuber (Mima), immature tuber (Baima), and mature tuber (Jianma) (Zhu et al., 2024). Among these, the mature tuber is the only commercially valuable harvest stage, and bolting is the critical turning point for its quality degradation. This study focuses on three key nodes before and after bolting of the mature tuber—pre-bolting tuber, post-bolting tuber, and stem. The pre-bolting tuber represents the optimal harvest period with the highest quality (Zhang et al., 2011; Zhu et al., 2024); although the post-bolting tuber shows diminished quality, metabolomic studies have revealed that it still possesses significant metabolic activity with numerous differential metabolites (Wang, 2024; Wang et al., 2024a), thus offering potential for resource reuse; the stem, as a direct product of bolting, has been shown to contain various active components in recent studies, with compounds such as kuwanon C and mornigrol H isolated from *G. elata* stems for the first time (Qin et al., 2023). These findings indicate that the stem has potential medicinal value worthy of further investigation.

Although the genetic mechanisms regulating bolting and flowering in the model plant *Arabidopsis* have been well characterized (Berentsen et al., 2024), the regulation of bolting and its metabolic consequences exhibit species-specificity across different plants. For medicinal plants where underground parts serve as the medicinal organ, bolting often induces significant changes in metabolite levels, thereby affecting medicinal quality. Studies have shown that bolting reduces the accumulation of ferulic acid and flavonoids in *Angelica sinensis*, while increasing lignin accumulation and inducing root lignification (Li et al., 2022). After bolting, the abundance of secondary metabolites in *Angelica dahurica*, including flavonoids, coumarins, and amino acids, significantly decreases (Li et al., 2025). Yuan et al (Yuan et al., 2020). reported that temperature and variety could influence the bolting characteristics of *G. elata*, suggesting that seeds of *G. elata* f. *elata* and *G. elata* f. *glauca* should be directly cultivated at a constant temperature of 22 °C for bolting production. Meanwhile, Wang et al (Wang et al., 2024). demonstrated that light quality significantly affects the bolting characteristics of *G. elata* f. *glauca*, with blue light being the optimal light quality during the bolting period. Notably, previous studies have primarily focused on the regulatory effects of environmental factors (such as temperature and light quality) on the phenotypic characteristics of *G. elata* bolting, representing explorations at the level of cultivation physiology. However, these studies failed to reveal the reprogramming mechanisms of the internal metabolic network during bolting, nor could they elucidate the molecular basis of active component accumulation at different developmental stages. In contrast, this study employs, for the first time, untargeted metabolomics to comprehensively analyze the dynamic metabolic changes in three key developmental stages of *G. elata*—pre-bolting tubers, post-bolting tubers, and stems—from a systems biology perspective. The objectives are to: (1) construct the metabolic regulatory network of the bolting process in *G. elata*; (2) clarify the accumulation patterns of key pharmacologically active components such as gastrodin and parishin C; and (3) identify specific metabolic markers for distinguishing different *G. elata* varieties.

Based on the above research background, this study proposes the following testable scientific hypotheses: (1) The bolting process of *Gastrodia elata* is accompanied by significant metabolic reprogramming, and the categories of metabolites undergo substantial changes; (2) The contents of the key pharmacologically active components, gastrodin and parishin C, change significantly during the bolting process of *Gastrodia elata*; (3) The contents of plant hormones in the three *Gastrodia elata* varieties change significantly across the three developmental stages. To test these hypotheses, three representative varieties of *Gastrodia elata*—*G. elata* f. *elata*, *G. elata* f. *glauca*, and dark red *G. elata*—were selected as research subjects. All three varieties are the main cultivated varieties in Xiaocaoba Town, Yiliang County, Zhaotong City, Yunnan Province, with extensive cultivation and significant economic value. Among them, *G. elata* f. *elata* and *G. elata* f. *glauca* are common varieties documented in the Chinese Pharmacopoeia, with distinct genetic backgrounds (Li et al., 2020; Liu et al., 2023), making them representative varieties of medicinal *Gastrodia elata* (Li et al., 2020); dark red *G. elata* is a recently discovered local specialty characterized by its blood-red pedicel, which may possess unique medicinal value (Wang et al., 2024b), and limited research is available to date. These three varieties were selected to compare the metabolic differences among *Gastrodia elata* varieties with different genetic backgrounds during key developmental stages using untargeted metabolomics, aiming to provide a basis for variety identification and resource utilization.

## 2 Materials and methods

### 2.1 Plant material and sample collection

**Figure 1:**
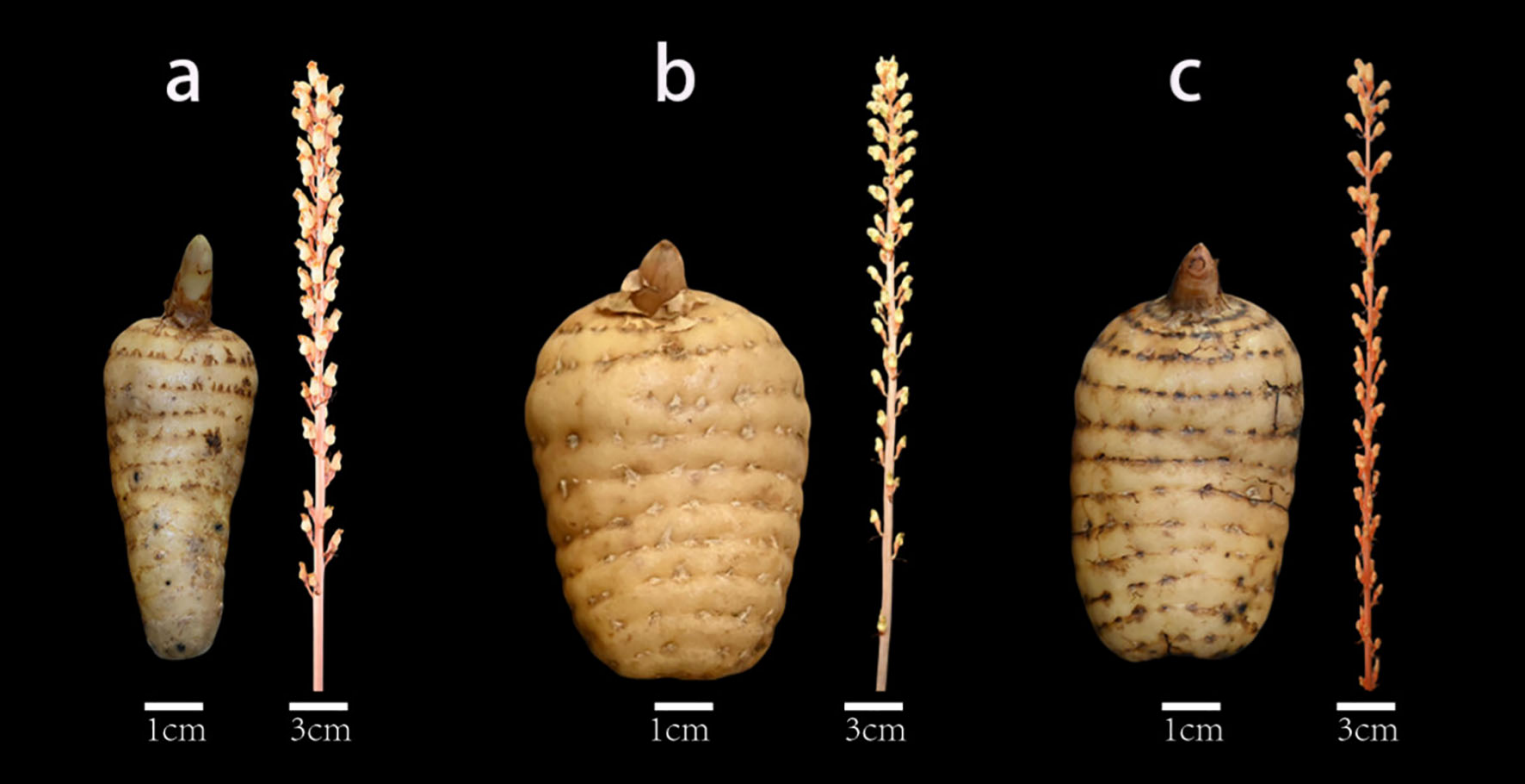
Illustrations of three *Gastrodia elata* varieties. **(a)** *G. elata* f. *elata* (Hong Tianma); **(b)** *G*. *elata* f. *glauca* (Wu Tianma); **(c)** dark red *G. elata* (XueHong Tianma).

Three varieties of *Gastrodia elata* were used as experimental materials in this study: Hong Tianma, the original form of *G. elata*, with the scientific name *Gastrodia elata* f. *elata*; Wu Tianma, a formal form of *G. elata*, with the scientific name *Gastrodia elata* f. *glauca*; and XueHong Tianma, which has not yet been formally published as an independent form or variety; therefore, we refer to it as “dark red *G. elata*” throughout the manuscript following the literature (Hu et al., 2025). The three varieties of *Gastrodia elata* were all collected from Xiaocaoba Town, Yiliang County, Zhaotong City, Yunnan Province (27.78° N, 104.52° E) (**Figure 1**) on March 28, 2024, and identified by Researcher Ji Pengzhang from the College of Traditional Chinese Medicine, Yunnan University of Chinese Medicine. The identification was primarily based on the morphological characteristics (Zeng et al., 2023; Jiang et al., 2026) and key agronomic traits of the three *Gastrodia elata* varieties: *G. elata* f. *elata* (Hong Tianma) has dumbbell-shaped tubers, a yellowish-brown epidermis, an orange-red stem, and an early bolting stage (early April); *G. elata* f. *glauca* (Wu Tianma) has elliptical to ovoid-elliptical tubers, a grayish-brown epidermis, a gray-brown stem with white longitudinal stripes, and a late bolting stage (mid-April); dark red *G. elata* (XueHong Tianma) has tuber morphology intermediate between the two, a light yellowish-brown epidermis, a deep red stem, and an intermediate bolting stage (early to mid-April). Jianma with a single tuber weight of 160-250g, free from mechanical damage, pests, and diseases, and exhibiting intact and robust morphology were selected as experimental materials. The experiment was conducted from March 29 to April 30, 2024. Cultivation containers were 37 cm × 28 cm foam boxes, and the growth substrate was sterilized sand and soil (sand: soil=1:1) with a pH of 5.5–6.0. During the cultivation period, the indoor temperature was controlled at 15-25 °C (20-25 °C during the day, 15-18 °C at night); soil moisture was strictly maintained at 50-60% using a hygrometer; relative air humidity was maintained at 70-80% with daily misting; light conditions consisted of natural indoor light without direct sunlight, with approximately 70% shading and a light intensity of approximately 200-300 μmol·m^-^²·s^-^¹, following the natural photoperiod (approximately 12 h light/12 h dark); windows were opened twice a day for 30 minutes each to maintain air circulation; and sterile water was used for irrigation every 2–3 days to maintain stable soil moisture. The sampling method was as follows: After collecting Gastrodia samples, surface soil was washed off, and a 1cm² tissue block was excised 1cm above the basal depression to serve as pre-bolting tuber samples for the three Gastrodia varieties. To prevent tuber rotting, the epidermis was retained, a high-fat membrane was evenly applied to the sampled area, and the area was then covered with the gastrodia epidermis. After the bolting of *Gastrodia elata*, the portion of the tuber approximately 1 cm from the apical bud and the central section of the stems with uniform height were collected as post-bolting tuber and stem samples, respectively. All samples were immediately frozen in liquid nitrogen after collection and stored at -80 °C for subsequent use. Three biological replicates were set up for each experiment, and the sampling information is shown in **Table 1**.

**Table 1:** Developmental stages of three *Gastrodia elata* varieties and their corresponding codes.

Developmental stage	Code
*G. elata* f. *elata* tuber before bolting	T-a-H
*G. elata* f. *glauca* tuber before bolting	T-a-W
dark red *G. elata* tuber before bolting	T-a-XH
*G. elata* f. *elata* tuber after bolting	T-b-H
*G. elata* f. *glauca* tuber after bolting	T-b-W
dark red *G. elata* tuber after bolting	T-b-XH
*G. elata* f. *elata* stem	S-H
*G. elata* f. *glauca* stem	S-W
dark red *G. elata* stem	S-XH

### 2.2 Metabolite extraction and metabolomics analysis

Metabolite extraction was performed according to the method described in reference (Zhu et al., 2025). The following procedures were applied: 60 mg of sample was placed into a 1.5 mL EP tube, and two small steel balls and 600 μL of pre-cooled methanol-water (4:1, v/v, containing 4 μg/mL mixed internal standard) were added. The sample was pre-cooled at -40 °C for 2 min, and then ground in a grinder (45 Hz, 2 min). Subsequently, ultrasonic extraction was performed in an ice-water bath for 30 min, followed by overnight standing at -40 °C. The next day, centrifugation was carried out at 12,000 rpm and 4 °C for 20 min, and 150 μL of the supernatant was transferred into an LC-MS injection vial equipped with a micro-insert for analysis. Quality control (QC) samples were prepared by mixing equal volumes of extracts from all samples. All extraction reagents were pre-cooled at -20 °C before use.

Chromatographic conditions were as follows: The extracts were analyzed using a Waters ACQUITY UPLC I-Class Plus/Thermo QE system (Waters Corporation/Thermo Fisher Scientific, MA, United States) equipped with an ACQUITY UPLC HSS T3 column (100 mm × 2.1 mm, 1.8 μm; Waters Corporation, Milford, MA, USA). The column temperature was maintained at 45 °C. The mobile phase consisted of A (water containing 0.1% formic acid) and B (acetonitrile). The flow rate was 0.35 mL/min, and the injection volume was 3 μL.

Mass spectrometry conditions were set as follows: Metabolites were identified by comparing retention time, accurate mass, and MS/MS fragmentation patterns with reference standards and public databases, including HMDB, Lipidmaps, METLIN, etc. Differentially accumulated metabolites (DAMs) were identified based on the criteria of VIP > 1.5 and *P* < 0.05.

### 2.3 Metabolic pathway analysis

Analysis of key pathways in three developmental stages of *Gastrodia elata*: KEGG enrichment analysis was performed based on the KEGG database (https://www.genome.jp/kegg/). The hypergeometric test was used to calculate pathway enrichment significance, and pathways with p-value ≤ 0.05 were considered significantly enriched.

Analysis of energy metabolic networks in three key developmental stages of *Gastrodia elata*: Target metabolites were imported into the MetaboAnalyst website (https://www.metaboanalyst.ca/) for KEGG enrichment analysis. The hypergeometric test was used to calculate the enrichment significance for each pathway, and P-values were adjusted using the Benjamini-Hochberg method to control the false discovery rate (FDR). Pathways with FDR < 0.05 were considered significantly enriched, and the final energy metabolic network was determined based on shared metabolites among pathways.

### 2.4 Parameter settings of the WGCNA-based metabolite co-expression network

Network construction was performed using the Pearson correlation coefficient to calculate correlations between metabolites. The soft threshold (power) was determined to be 20 based on scale-free network fit, with a signed hybrid network type. Module identification was performed using the dynamic tree cutting method with a minimum module size of 30, and modules with eigengene correlations > 0.75 were merged (merging threshold of 0.25).

### 2.5 Criteria for highlighted metabolite selection

The metabolites highlighted in this study were selected based on the following criteria: (1) Known bioactive components: gastrodin and parishin C are the main active components of *G. elata* and were included based on the Chinese Pharmacopoeia and literature reports; (2) Statistical significance: phytohormones such as jasmonic acid and gibberellins showed significant differences before and after bolting (VIP > 1.5, *P* < 0.05) and were enriched in key pathways identified by KEGG enrichment analysis; (3) WGCNA analysis: core metabolites in modules highly correlated with target bioactive components (|r| > 0.8); (4) Literature support: previous studies have reported their involvement in plant bolting/flowering.

### 2.6 Statistical analysis

One-way analysis of variance (ANOVA) was performed using IBM SPSS Statistics 26.0 (Armonk, NY, United States), with Tukey’s honestly significant difference (HSD) test employed to determine significant differences. Hierarchical cluster analysis (HCA) plots, principal component analysis (PCA) plots, pie charts, bar charts, and line charts were created using OriginPro 2024 software (Hampton, MA, United States). KEGG bubble plots were generated using the OECloud tool. Pathway diagrams were drawn using Adobe Illustrator 2024 (Adobe Inc., San Jose, CA, United States).

## 3 Result

In this study, we analyzed three *Gastrodia elata* varieties: *G. elata* f. *elata* (Hong Tianma), *G. elata* f. *glauca* (Wu Tianma), and dark red *G. elata* (XueHong Tianma).

### 3.1 Comprehensive metabolomic analysis of *Gastrodia elata* at three developmental stages

**Figure 2:**
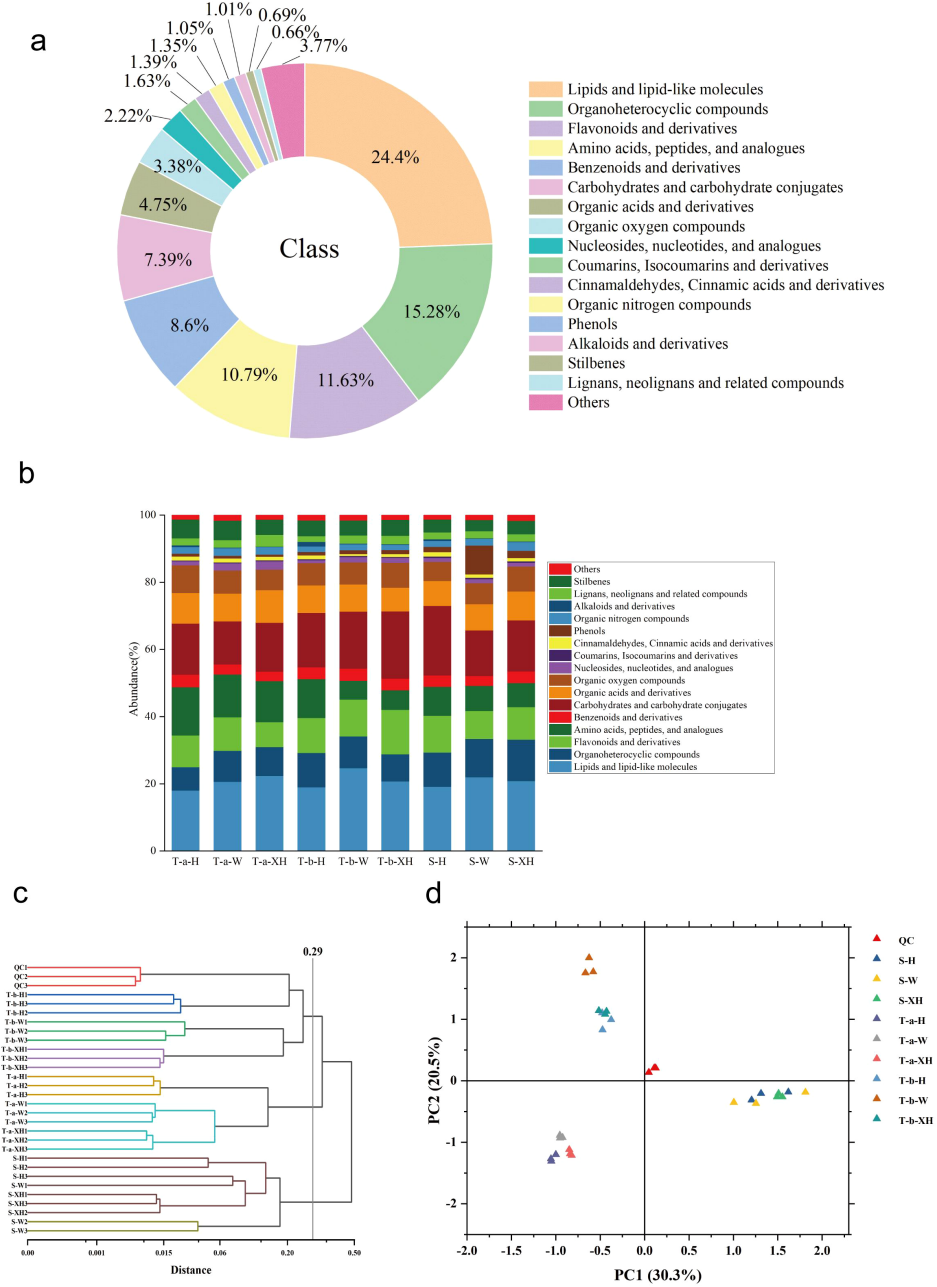
Metabolite analysis results of *Gastrodia elata* at three developmental stages: **(a)** Classification and proportion of annotated metabolites; **(b)** Stacked bar chart showing the average proportion of each metabolite class at three developmental stages (pre-bolting tuber, post-bolting tuber, and stem). The data represent the average proportion across all three varieties combined (*G. elata* f. *glauca*, *G. elata* f.*elata*, and dark red *G. elata* for each developmental stage. The y-axis represents the percentage of total metabolite abundance; **(c)** Hierarchical cluster analysis (HCA) plot, and **(d)** Principal component analysis (PCA) plot.

To investigate the dynamic changes in metabolite types and relative levels during the development of three *Gastrodia elata* varieties, we performed non-targeted metabolomics analysis on pre-bolting tubers, post-bolting tubers, and stems. We identified a total of 6,209 metabolites and classified them into four confidence levels according to the Metabolomics Standards Initiative (MSI) guidelines: Level 1 (410 metabolites), Level 2 (1,290 metabolites), Level 3 (1,066 metabolites), and Level 4 (3,443 metabolites). The total metabolites covered 17 categories (**Figure 2a**). This includes 1,515 lipid and lipid-like molecules, 949 organic heterocyclic compounds, 722 flavonoids and their derivatives, 670 amino acids, peptides, and analogues, 534 benzene and its derivatives, 459 carbohydrates and carbohydrate conjugates, 295 organic acids and their derivatives, 210 organic oxygen compounds, 138 nucleosides, nucleotides, and analogues, 101 coumarins, isocoumarins and their analogues, 86 cinnamaldehydes, cinnamic acids and their derivatives, 84 organic nitrogen compounds, 65 phenols, 63 alkaloids and their derivatives, 43 stilbenes, 41 lignans, neolignans and related compounds, and 234 other compounds. During the bolting process of *Gastrodia elata*, the proportions of stilbenes, organic oxygen compounds, and amino acid compounds gradually decrease, while the proportions of phenols and carbohydrates progressively increase (**Figure 2b**). The principal component analysis (PCA) plot showed that the quality control samples (QC) clustered closely at the center (**Figure 2d**). To objectively evaluate the stability and reproducibility of the experiment, we calculated the relative standard deviation (RSD) of peak areas for all metabolites in QC samples. The results showed that the RSD values of all metabolites were below 30%, indicating good stability and reproducibility of the metabolomic data. The hierarchical clustering analysis (HCA) dendrogram revealed that the samples could be divided into three groups at a Euclidean distance of 0.29: the stem group, the pre-bolting tuber group, and the post-bolting tuber group (**Figure 2c**). This was consistent with the PCA results, demonstrating that the variation between developmental stages was far greater than that between varieties. The differential metabolites at each developmental stage were significantly different (*P* < 0.05). To quantify the contributions of developmental stage and variety, PERMANOVA analysis (based on Bray-Curtis distance with 999 permutations) was performed. The results showed (**Supplementary Table 1**) that developmental stage explained 63.46% of the total variance (R² = 0.6346, *P* < 0.001), while variety explained 14.25% of the total variance (R² = 0.1425, *P* < 0.001). This quantitative analysis confirms that developmental stage is the dominant factor driving metabolomic variation in *G. elata*, contributing approximately 4.4 times more (63.5%) than variety (14.3%).

### 3.2 Total active components and amino acid profiles of *Gastrodia elata* at three developmental stages

**Figure 3:**
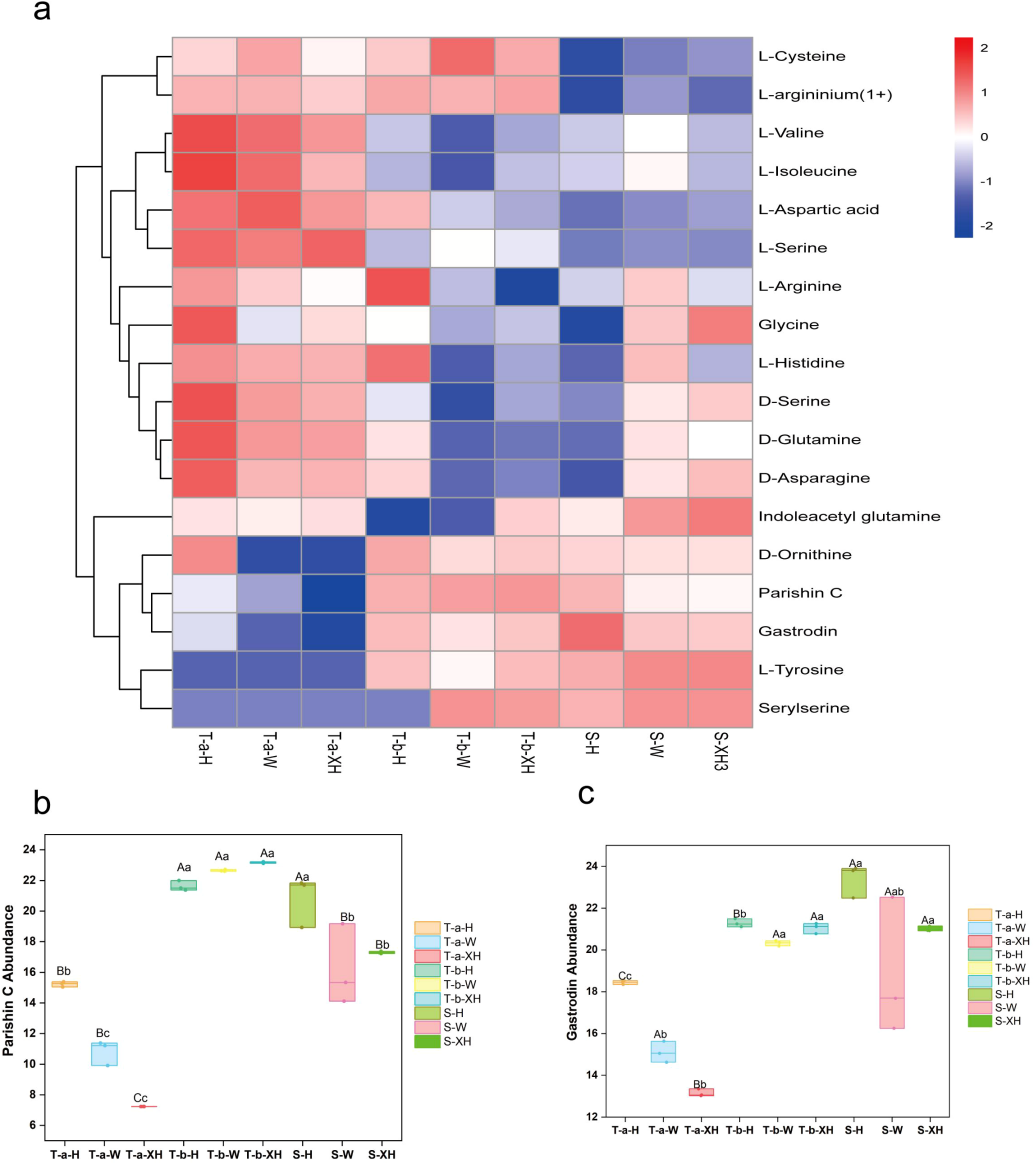
Total active components and amino acid profiles of *Gastrodia elata* at three developmental stages: **(a)** Cluster heatmap of amino acids, parishin C, and gastrodin in three *Gastrodia elata* varieties: the heatmap displays Z-score normalized abundance values, colors represent metabolite expression levels ranging from low (blue) to high (red); **(b)** Box plot of parishin C abundance; **(c)** Box plot of gastrodin abundance. Letter labels in **(b)** and **(c)** indicate significant differences between developmental stages within the same variety, as determined by one-way ANOVA followed by Tukey’s Honestly Significant Difference (HSD) post-hoc test. Uppercase letters denote significance at *P* < 0.01, and lowercase letters at *P* < 0.05.

We identified seven active components (Zhu et al., 2024) and amino acids in three *Gastrodia elata* varieties across three developmental stages. However, only gastrodin and parishin C were consistently detected and reached the quantification limit in all samples, while the other five components were not detected or fell below the detection limit. Additionally, we identified 16 amino acids. Therefore, this section presents only the content changes of gastrodin and parishin C across the three developmental stages, along with the overall distribution of amino acids (**Figure 3a**). Results showed that most amino acids exhibited higher abundance in tubers before bolting, with decreased levels post-bolting. In contrast, both active components (gastrodin and parishin C) along with three amino acids (D-Ornithine, L-Tyrosine, and Serylserine) demonstrated lower abundance prior to bolting and increased abundance after bolting. We observed that the abundance of parishin C was highest in the tubers after bolting, followed by the stems, and lowest in the tubers before bolting. The abundance of parishin C in the tubers after bolting was significantly higher than that in the tubers before bolting across all three *Gastrodia elata* varieties (*P* < 0.01). Gastrodin exhibited the highest abundance in the stems, followed by the tubers after bolting, and was lowest in the tubers before bolting. In both Hong Tianma and XueHong Tianma, the abundances of parishin C and gastrodin in the stems were significantly higher than those in the tubers before bolting (*P* < 0.01), while in Wu Tianma, the abundances of parishin C and gastrodin in the stems were significantly higher than those in the tubers before bolting (*P* < 0.05) (**Figures 3b, c**; **Supplementary Table 2**). (The p-values presented in this section were obtained after one-way ANOVA followed by Tukey’s HSD post-hoc test for multiple comparisons correction).

### 3.3 WGCNA analysis of active components and metabolites in key developmental stages of *Gastrodia elata*

**Figure 4:**
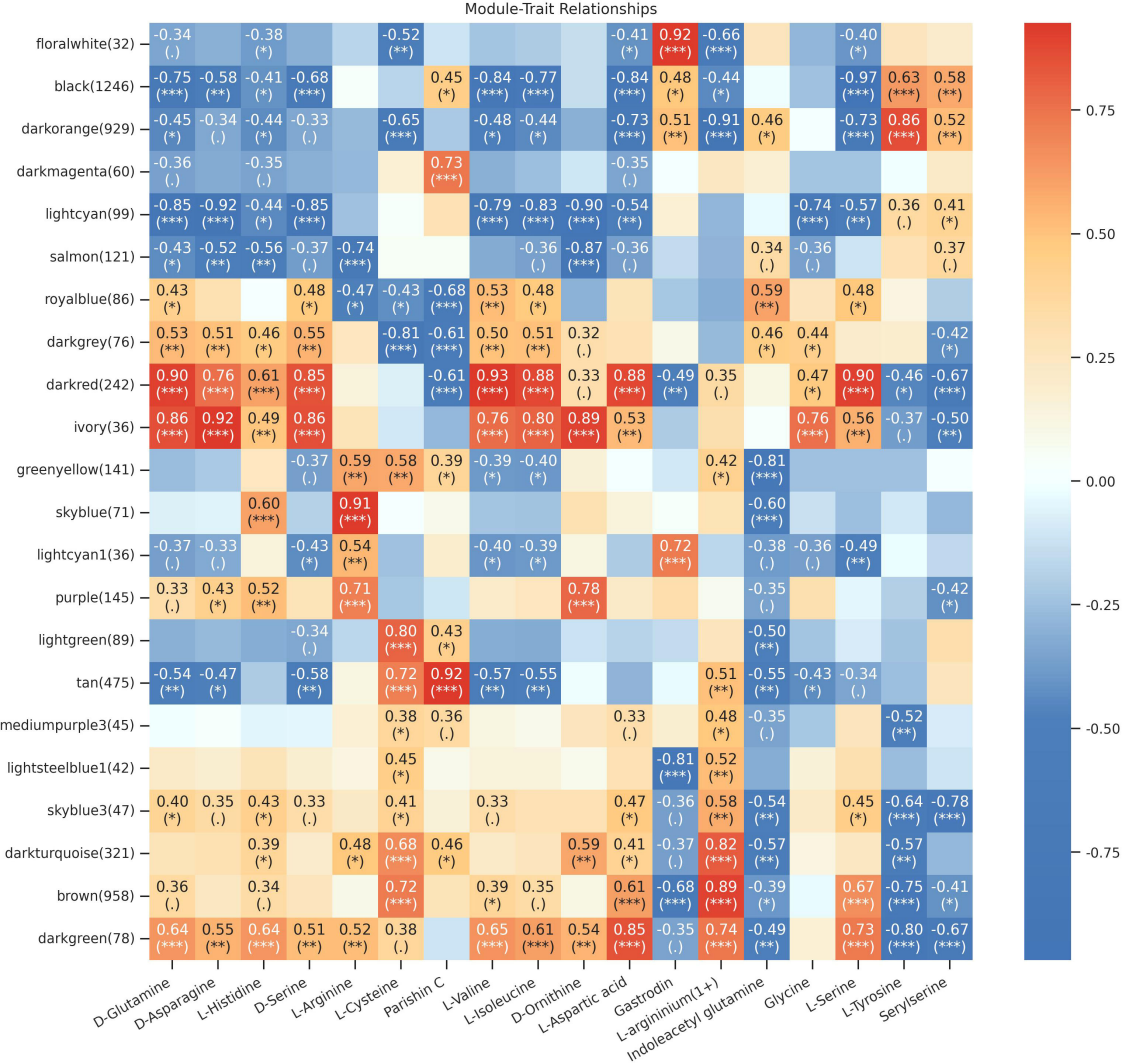
Heatmap of correlation coefficients between active components of *Gastrodia elata* and WGCNA modules. Shades approaching red indicate positive correlations, while shades approaching blue indicate negative correlations. The numbers in each square represent the correlation coefficients between modules and active components. Values closer to 1 indicate stronger positive correlations, while values closer to -1 indicate stronger negative correlations. Symbols in parentheses denote significance levels, with * indicating *P* < 0.05, ** indicating *P* < 0.01, and *** indicating *P* < 0.001.

To identify metabolites closely associated with the active components (16 amino acids, gastrodin, and parishin C), we constructed a co-expression network using weighted gene co-expression network analysis (WGCNA) based on 5,524 metabolites (after filtering out low-variance metabolites with standard deviation ≤ 0.5) (**Figure 4**, **Supplementary Table 3**). Although WGCNA was originally developed for gene expression data, it has been widely applied in metabolomics research, as co-expression relationships among metabolites can effectively reflect coordinated changes within the same metabolic pathways or processes (Fu et al., 2025). According to the official recommendations of the WGCNA authors, a minimum of 15 samples is required for WGCNA analysis, and sample sizes exceeding 20 generally yield better performance with more reliable and robust results (Zhang et al., 2022). The 27 samples in this study (3 varieties × 3 developmental stages × 3 biological replicates) far exceed this minimum requirement and meet the basic conditions for WGCNA analysis. The sample size in this study is 27, with 5,524 metabolites, yielding a sample-to-metabolite ratio of approximately 1:205. Network robustness was ensured through strict parameter settings in this study. A total of 23 modules were ultimately obtained (**Supplementary Figure 1**), with the smallest non-grey module being the floralwhite module, containing 32 metabolites, exceeding the set minimum threshold. The grey module (unassigned metabolites) contained only 149 metabolites, accounting for 2.7% of the total, indicating good specificity in module assignment and demonstrating that the vast majority of metabolites were successfully assigned to biologically meaningful modules. The results showed that the dark red module was highly correlated with six amino acids (*r* > 0.85, *P* < 0.001), and the ivory module was highly correlated with five amino acids (*r* > 0.8, *P* < 0.001). The floralwhite module was highly correlated with gastrodin (*r* = 0.92), and within this module, (R)-mandelic Acid showed an even higher correlation with gastrodin (*r* = 0.94). The tan module demonstrated a significant correlation with parishin C (correlation coefficient *r* = 0.92), with the flavonoid glycoside Zeravschanoside reaching a correlation of 0.93 with parishin C. Further analysis of metabolite categories in the floralwhite and tan modules revealed that flavonoids were the predominant metabolites in both modules. We constructed network diagrams for the metabolites in these two modules (**Supplementary Figure 2**) to identify core metabolites. The results showed that in the floralwhite module, except for metabolites such as Triacetin and Hexadecadienylcarnitine, all other metabolites were highly interconnected. Among them, Isorhamnetin, (R)-mandelic Acid, and Tarenninoside E exhibited the highest connectivity with other metabolites in the module, and we identified them as core metabolites of the floralwhite module. In the tan module, metabolites such as Kanzonol B, 6-Thioinosine, and Austrobuxusin K displayed high connectivity with other metabolites and occupied central positions, and we identified them as core metabolites of this module.

### 3.4 Comparative analysis of differential metabolites in *Gastrodia elata* at three different developmental stages

**Figure 5:**
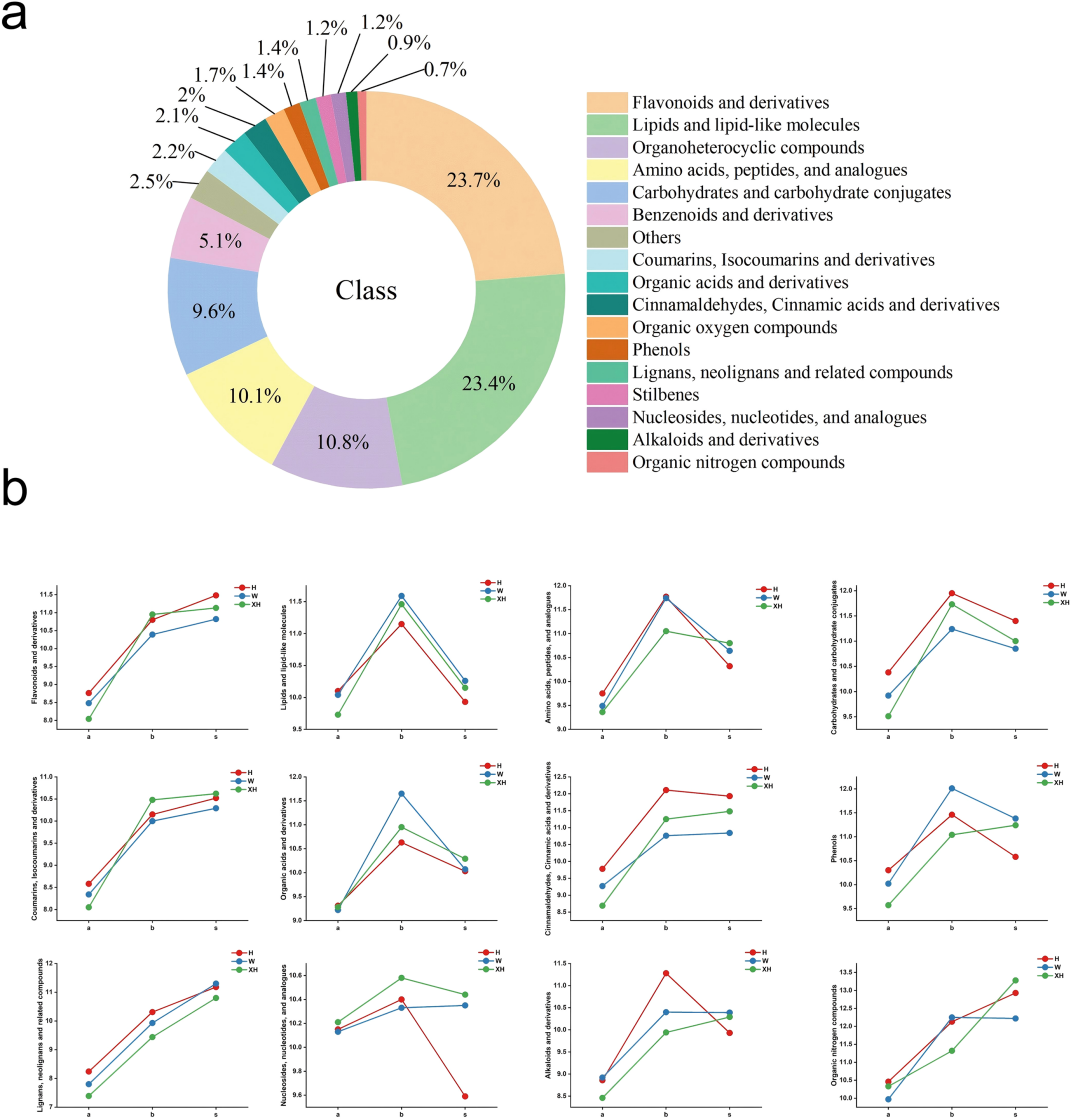
Results of differential metabolite analysis: **(a)** Classification and proportion of differential metabolites; **(b)** Line chart showing abundance variation trends of major differential metabolite categories across three developmental stages of *Gastrodia elata* (the x-axis represents developmental stages, where ‘a’ denotes pre-bolting tubers, ‘b’ indicates post-bolting tubers, and ‘s’ stands for stems; the y-axis represents different categories of metabolites. Data were analyzed using one-way ANOVA and Tukey HSD test. The legend indicates different *Gastrodia elata* varieties: H, Hong Tianma; W, Wu Tianma; XH, XueHong Tianma).

A total of 1,624 differential metabolites (VIP > 1.5, *P* < 0.05) were screened from tuber and stem samples of three Gastrodia varieties. Among these metabolites (**Figure 5a**), there are 380 lipids and lipid-like molecules, 175 organic heterocyclic compounds, 385 flavonoids and their derivatives, 164 amino acids, peptides, and analogues, 83 benzenoids and their derivatives, 156 carbohydrates and carbohydrate conjugates, 34 organic acids and their derivatives, 27 organooxygen compounds, 20 nucleosides, nucleotides, and analogues, 35 coumarins, isocoumarins, and their analogues, 33 cinnamaldehydes, cinnamic acids, and their derivatives, 12 organonitrogen compounds, 22 phenols, 15 alkaloids and their derivatives, 20 stilbenes, 22 lignans, neolignans, and related compounds, and 41 other compounds.

To evaluate the changes in differential metabolites of three Gastrodia varieties at different developmental stages, a one-way ANOVA was performed on the major categories of differential metabolites after data normalization using the following approach: For each biological replicate, the arithmetic mean abundance of all differential metabolites belonging to the same metabolite class was calculated to obtain a class-level mean abundance value. One-way ANOVA was then conducted with developmental stage (pre-bolting tubers, post-bolting tubers, and stems) as the fixed factor on these class-level mean abundances, followed by Tukey’s HSD test for multiple comparisons. The results showed that the abundances of flavonoids, coumarins, lignans, and organic nitrogen compounds peaked in the stems, while the abundances of lipids, organic acids, amino acids, and carbohydrates peaked in the tubers after bolting (**Supplementary Table 5**; **Figure 5b**).

### 3.5 Analysis of differential metabolites in tubers and stems at different developmental stages of three *Gastrodia elata* varieties

#### 3.5.1 The same *Gastrodia elata* at different developmental stages

To clarify the metabolic changes across different developmental stages of the same *Gastrodia elata* variety, we analyzed the metabolites in pre-bolting tubers, post-bolting tubers, and stems of each of the three varieties separately. The results showed that in both Hong Tianma and Wu Tianma, lipids were the predominant components in tubers before and after bolting, while flavonoids were the main constituents in stems. In XueHong Tianma, lipids primarily dominated the tubers before bolting, whereas flavonoids became the major components in tubers after bolting and in stems. We conducted cluster heatmap analysis on the common differential metabolites of lipids and flavonoids in tubers before bolting, tubers after bolting, and stems of each Tianma variety (**Supplementary Figure 3**).

##### 3.5.1.1 Cluster heatmap analysis of Hong Tianma across different developmental stages

The lipid clustering heatmap of Hong Tianma (**Supplementary Figure 3a**) revealed that 26 lipids, including Glycerophosphoinositol, 13S-HOTrE, and 8-deoxy-J2-IsoP, exhibited the highest abundance in tubers before bolting. Meanwhile, 28 lipids reached peak abundance in tubers after bolting, including 1 sterol, 1 sphingolipid, 4 glycerolipids, 9 prenol lipids, and 13 fatty acyls. Notably, MGMG (18:2/0:0), Gingerglycolipid A, and 15-Acetoxyscirpene-3,4-diol 4-O-a-D-glucopyranoside showed 1.76-, 1.86-, and 1.65-fold upregulation in post-bolting tubers compared to pre-bolting tubers, respectively. Three glycolipids (4-Hydroxy-5-(3’,5’-dihydroxyphenyl)-valeric acid glucuronide, 6-[5-(4-carboxy-2-hydroxybutyl)-2-hydroxyphenoxy]-3,4,5-trihydroxyoxane-2-carboxylic acid, and 3,4-Dihydroxyphenylvaleric acid 4 glucuronide) demonstrated the highest abundance in stems.

The flavonoid clustering heatmap (**Supplementary Figure 3b**) results revealed that flavonoid glycosides were the primary category influencing abundance variations during the developmental stages of Hong Tianma Fifteen differential metabolites, including Chrysosplenoside H, reached peak abundance in the tubers after bolting. Notably, 4’-Hydroxy-5,6,7-trimethoxyflavanone and Myricetin 3-(4’’-malonylrhamnoside) showed 2.05-fold and 1.91-fold upregulation in post-bolting tubers compared to pre-bolting tubers, respectively, while their abundance decreased in the stems.

##### 3.5.1.2 Cluster heatmap analysis of Wu Tianma across different developmental stages

The lipid clustering heatmap results of Wu Tianma (**Supplementary Figure 3c**) revealed that 36 lipid molecules, including Humulol, Lucidenic acid A, and Cinnzeylanol, exhibited the highest abundance in tubers before bolting. Meanwhile, 39 lipid molecules, including Gibberellin A8, reached peak abundance in the stems of Wu Tianma. The remaining lipid metabolites achieved their peak abundance in tubers after bolting.

The flavonoid clustering heatmap (**Supplementary Figure 3d**) revealed that flavonoid glycosides are the primary category affecting the abundance variation in Wu Tianma. The compounds that peaked in abundance in the tubers after bolting mainly included 11 flavonoid glycosides and 3 O-methylated flavonoids. Among them, the abundance of Catechin-(4alpha->8)-gallocatechin-(4alpha->8)-catechin and Liquiritigenin 7-glucoside-4’-apiosyl-(1->2)-glucoside in post-bolting tubers increased by over 1.6-fold compared to pre-bolting tubers, while their abundance decreased in the stems.

##### 3.5.1.3 Cluster heatmap analysis of XueHong Tianma across different developmental stages

The heatmap of lipid clustering in XueHong Tianma (**Supplementary Figure 3e**) revealed that 25 lipid molecules, including Lupeoside, were significantly highly expressed (*P* < 0.05) in pre-bolting tubers. Among these, sn-Glycero-3-phosphocholine, MG (0:0/18:3/0:0), and MG (18:1-O/0:0/0:0) accounted for 85.74% of the total abundance of the 25 lipids, indicating that these three lipids are the major lipid components in pre-bolting tubers of XueHong Tianma. Additionally, 26 lipid molecules, consisting of 1 glycerolipid, 2 prenol lipids, 3 sterols, and 20 fatty acyls, reached their highest abundance in post-bolting tubers. Notably, the abundance of 15S-HETrE and 11-deoxy-PGF2*α* in post-bolting tubers was upregulated by 1.41-fold and 1.42-fold, respectively, compared to pre-bolting tubers. Furthermore, 18 lipid molecules, including Gibberellin A8 and Gibberellin A75, exhibited their highest abundance values in the stems.

The flavonoid clustering heatmap (**Supplementary Figure 3f**) revealed that the metabolites peaking in tuber abundance after bolting of XueHong Tianma primarily included 17 flavonoid glycosides (including Quercetin 3-(6’’-malonylglucoside)) and 4 O-methylated flavonoids. Among these, Butein 4’-arabinosyl-(1->4)-galactoside and Liquiritigenin 7-glucoside-4’-apiosyl-(1->2)-glucoside showed 1.95-fold and 1.94-fold upregulation respectively in post-bolting tubers compared to pre-bolting tubers, while their abundance decreased in stems.

#### 3.5.2 Different *Gastrodia elata* plants at the same developmental stage

**Figure 6:**
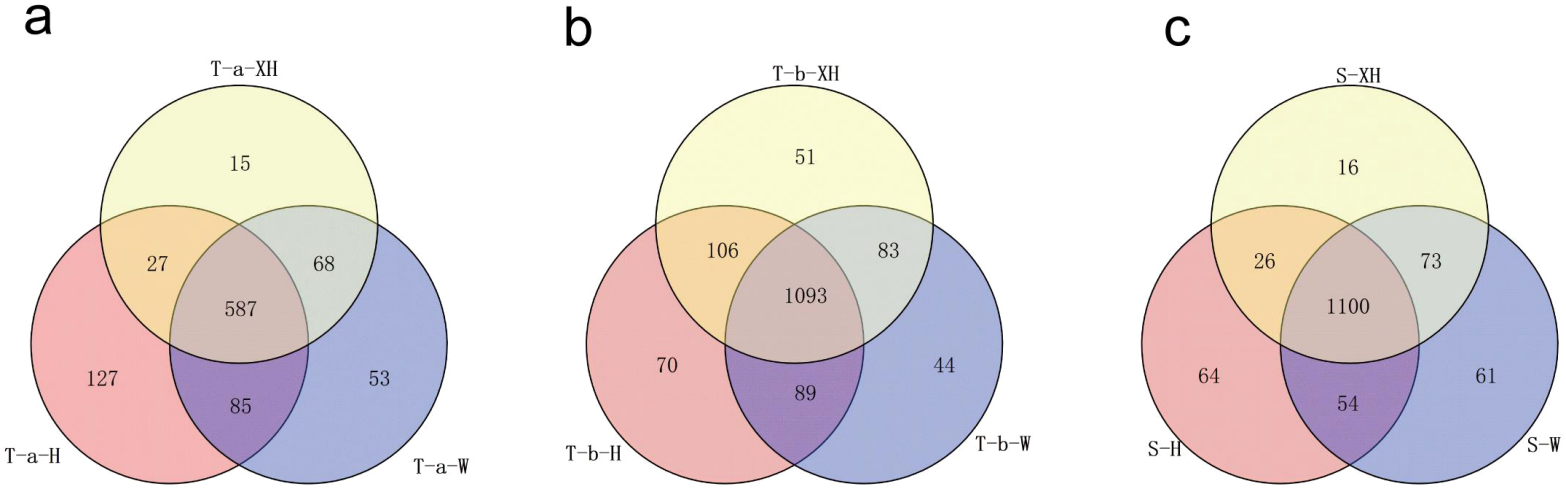
Venn diagram results of three developmental stages of *Gastrodia elata*: **(a)** Venn diagram results of tubers before bolting, **(b)** Venn diagram results of tubers after bolting, **(c)** Venn diagram results of stems.

To clarify the metabolite categories in tubers before bolting, tubers after bolting, and stems of three *Gastrodia elata* varieties, we conducted a Venn diagram analysis of the same developmental stage for the three varieties (**Figure 6**).

The Hong Tianma tuber before bolting had 127 unique differential metabolites, with flavonoids being the major compound group (40 species, 31.4%), and flavonoid glycosides as the primary subclass (25 species, 62.5%). The Wu Tianma tuber before bolting contained 53 unique differential metabolites, with flavonoids as the main compound group (14 species, 26.4%), and flavonoid glycosides as the dominant subclass (5 species, 35.7%). The XueHong Tianma tuber before bolting showed 15 unique differential metabolites, primarily amino acids (5 species, 33.3%). The three cultivars of *Gastrodia elata* tubers before bolting shared 196 common differential metabolites, mainly lipids (33.3%), with fatty acyls being the predominant subclass (113 species, 57.6%) (**Figure 6a**).

The Hong Tianma tuber after bolting had 70 unique differential metabolites, with flavonoids and lipids being the major compound classes (30 species, 42.8%), and flavonoid glycosides and isopentenol lipids as the main subclasses (17 species, 56.6%). The Wu Tianma tuber after bolting contained 44 unique differential metabolites, with lipids as the predominant compound class (9 species, 20.4%) and isopentenol lipids as the main subclass (5 species, 55.5%). The XueHong Tianma tuber after bolting showed 51 unique differential metabolites, with flavonoids being the major compound class (20 species, 39.2%) and flavonoid glycosides as the primary subclass (11 species, 55.0%). The three cultivars of *Gastrodia elata* tubers after bolting shared common differential metabolites mainly consisting of lipids (274 species, 25.0%), with fatty acyls being the dominant subclass (142 species, 51.8%) (**Figure 6b**).

The Hong Tianma stems contain 64 unique differential metabolites, with flavonoids being the major compound class (15 species, 23.4%), and flavonoid glycosides as the primary subclass (10 species, 66.6%). The Wu Tianma stems possess 61 unique differential metabolites, dominated by lipid compounds (29 species, 47.5%), with fatty acyls as the main subclass (14 species, 48.2%). The XueHong Tianma stems exhibit 16 unique differential metabolites, primarily composed of lipid compounds (6 species, 37.5%), including 2 sterols, 2 prenol lipids, and 2 fatty acyls. The common differential metabolites in the three cultivars of *Gastrodia elata* stems were primarily flavonoids (285 species, 25.9%), with flavonoid glycosides being the predominant subclass (169 species, 59.2%) (**Figure 6c**).

Additionally, 22 metabolites were identified as unique to Hong Tianma (i.e., metabolites not detected in any developmental stage of the other two varieties), 18 metabolites were unique to Wu Tianma, and 3 metabolites were unique to XueHong Tianma (**Supplementary Table 5**). These metabolites may serve as distinctive markers for identifying the three *Gastrodia elata* varieties.

### 3.6 Analysis of key pathways in three developmental stages of *Gastrodia elata*

**Figure 7:**
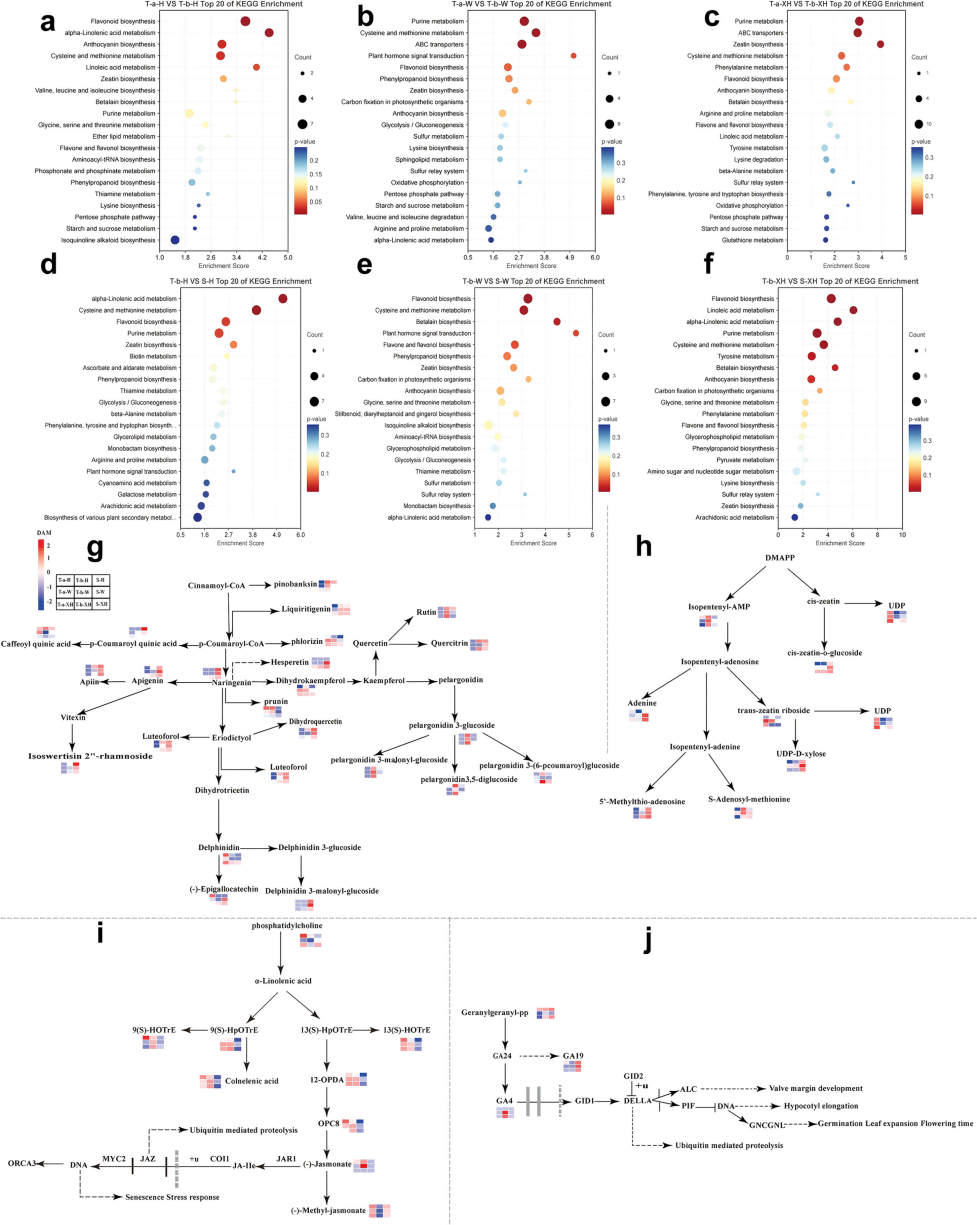
Pathway analysis of three Gastrodia development stages. **(a, d)** KEGG enrichment analysis of Hong Tianma development stage; **(b, e)** KEGG enrichment analysis of Wu Tianma development stage; **(c, f)** KEGG enrichment analysis of XueHong Tianma development stage; **(g)** Flavonoid biosynthesis pathway; **(h)** Zeatin biosynthesis pathway; **(i, j)** Plant hormone signal transduction pathway.

To elucidate the core pathways involved in the growth and development of *Gastrodia elata*, KEGG enrichment analysis of differential metabolites was conducted (**Figures 7a–f**; **Supplementary Table 6**). The results showed that the primary pathways influencing the developmental stages of Hong Tianma were flavonoid biosynthesis, *α*-linolenic acid metabolism, and cysteine and methionine metabolism; for Wu Tianma, the main pathways were cysteine and methionine metabolism and flavonoid biosynthesis; for XueHong Tianma, the key pathways included purine metabolism, zeatin biosynthesis, cysteine and methionine metabolism, tyrosine metabolism, and betalain biosynthesis. These pathways exhibited the most significant enrichment during the developmental stages of the three *Gastrodia elata* varieties (*P* < 0.05). Further research on how flavonoid biosynthesis-related pathways, zeatin biosynthesis pathways, and plant hormone signal transduction pathways affect the growth and development of three Gastrodia varieties revealed that a total of 24 differential metabolites were enriched in flavonoid biosynthesis-related pathways (**Figure 7g**). Among these, the majority of flavones and flavonols were accumulated in the stems. In Hong Tianma, the abundances of Apiin, Isowertin 2’’-rhamnoside, and Delphinidin 3-(6’’-malonylglucoside) were upregulated by 1.90, 1.98, and 1.54 times respectively compared to the pre-bolting tubers; while in Wu Tianma, the abundances of Hesperetin and Delphinidin 3-(6’’-malonylglucoside) were upregulated by 1.66 and 1.76 times respectively compared to the pre-bolting tubers. We found that Rutin and four types of pelargonidin (pelargonidin 3-glucoside, pelargonidin 3-malonyl-glucoside, pelargonidin 3,5-diglucoside, and pelargonidin 3-(6-pcoumaroyl) glucoside) were all accumulated in the tubers after bolting. The abundance of rutin in Hong Tianma, Wu Tianma, and XueHong Tianma tubers increased by 1.60, 1.71, and 1.80 times respectively compared to that before bolting. In Hong Tianma, the abundance of Callistephin was upregulated by 1.58-fold compared to pre-bolting tubers; in Wu Tianma, the abundance of Pelargonidin 3-(6’’-malonylglucoside) was upregulated by 1.66-fold compared to pre-bolting tubers; in XueHong Tianma, the abundances of Callistephin, Pelargonidin 3-(6’’-malonylglucoside), Pelargonin, and Pelargonidin 3-p-coumarylglucoside were upregulated by 1.60, 1.58, 1.71, and 1.76-fold respectively compared to pre-bolting tubers.

Zeatin is an important plant hormone that plays a crucial role in plant growth and development. This study identified eight differential metabolites involved in the zeatin biosynthesis pathway (**Figure 7h**). Among them, N6-(delta2-isopentenyl)-adenosine 5’-monophosphate and S-Adenosylmethionine were accumulated in tubers after bolting. Specifically, the abundance of N6-(delta2-isopentenyl)-adenosine 5’-monophosphate in Wu Tianma and XueHong Tianma tubers increased by 1.56-fold and 1.69-fold, respectively, after bolting compared with pre-bolting tubers. Meanwhile, the abundance of S-Adenosylmethionine in Hong Tianma tubers showed a 1.66-fold increase in post-bolting tubers compared to pre-bolting tubers. Four metabolites including Adenine were accumulated in the stems, while trans-zeatin riboside monophosphate and Uridine 5’-diphosphate were consumed during the bolting process.

The plant hormone signal transduction pathway primarily involves two pathways associated with α-linolenic acid metabolism and diterpenoid biosynthesis (**Figures 7i, j**). This study identified 9 differential metabolites related to the α-linolenic acid metabolic pathway, among which 5 showed similar expression patterns, all exhibiting significantly downregulated abundance in stems (*P* < 0.05). We observed that in Wu Tianma, jasmonic acid displayed a 1.69-fold significant upregulation in tubers after bolting compared to pre-bolting tubers, while no significant abundance changes were detected in Hong Tianma and XueHong Tianma. Therefore, jasmonic acid may be associated with the bolting process in Wu Tianma.

This study focused on the abundance changes of GA4 and GA19 (**Figure 7j**) during the developmental stages of the three *Gastrodia elata* varieties. The results showed that GA4 abundance in Wu Tianma was significantly upregulated by 1.69-fold in post-bolting tubers compared to pre-bolting tubers (*P* < 0.01), GA19 abundance in Wu Tianma stems was upregulated by 1.44-fold compared to pre-bolting tubers, and GA4 abundance in XueHong Tianma was upregulated by 1.49-fold in post-bolting tubers compared to pre-bolting tubers. This may indicate that increased gibberellin abundance promotes bolting in *Gastrodia elata*.

### 3.7 Analysis of energy metabolic networks in three key developmental stages of *Gastrodia elata*

**Figure 8:**
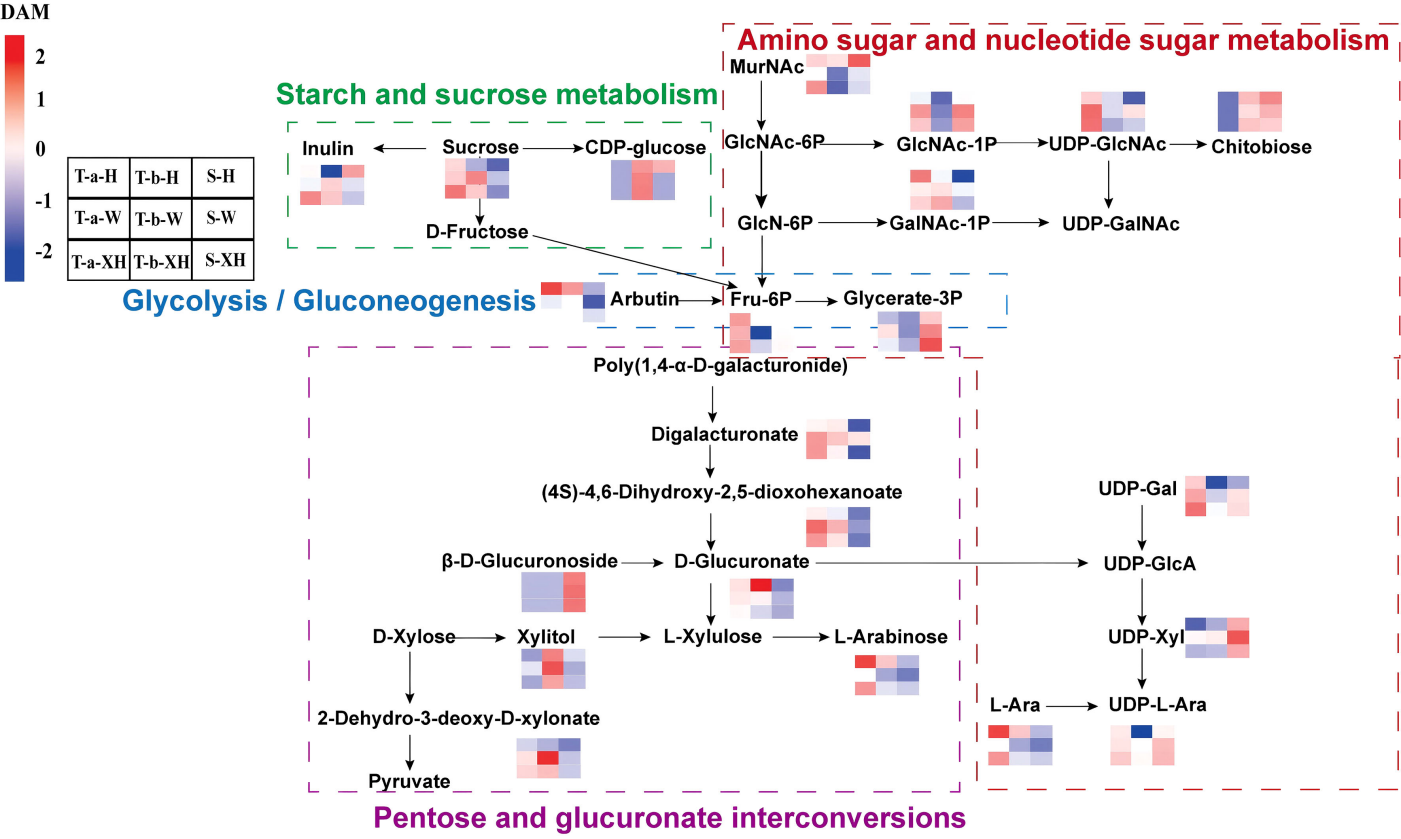
Energy metabolic pathways of *Gastrodia elata* at three developmental stages.

To elucidate the energy metabolism processes involved in the critical developmental stages of three Gastrodia varieties, we conducted KEGG enrichment analysis on all lipids, amino acids, carbohydrates, and nucleotides, identifying a total of 73 enriched pathways. The MetaboAnalyst website revealed significantly enriched pathways, including amino sugar and nucleotide sugar metabolism (**Supplementary Figure 4**). Three intersecting pathways were identified: starch and sucrose metabolism, glycolysis/gluconeogenesis, and pentose and glucuronate interconversions (**Figure 8**). The pathways are interconnected through shared metabolites: Fru-6P is a shared metabolite of glycolysis/gluconeogenesis, starch and sucrose metabolism, and amino sugar and nucleotide sugar metabolism; D-Glucuronate is a shared metabolite of pentose and glucuronate interconversions and amino sugar and nucleotide sugar metabolism. Together, they constitute the energy metabolic network diagram during the bolting stage of the three *Gastrodia elata* varieties. The results showed that the abundance of metabolites such as sucrose, UDP-GlcNAc, Fru-6P, L-Arabinose, and UDP-Gal decreased after bolting, suggesting that these metabolites may serve as energy reserves for plant bolting. Notably, CDP-glucose and xylitol exhibited the highest abundance in tubers after bolting but decreased in stems, indicating these two metabolites might provide energy for sustained stem growth. Meanwhile, Glycerate-3P, *β*-D-Glucuronoside, and UDP-Xyl were only highly expressed in stems.

## 4 Discussion

### 4.1 Bioactive components in *Gastrodia elata* stems and their potential applications

Gastrodin is the most abundant active monomeric component in *Gastrodia elata*, exhibiting remarkable anti-inflammatory, antioxidant, and neuroprotective effects. It is widely used in the treatment of various diseases including central nervous system disorders, cardiovascular diseases, and endocrine conditions (Wang et al., 2024; Wang et al., 2024). Parishins demonstrate potent anti-aging properties. Research by Gong et al (Gong et al., 2023). indicates that Parishins can prevent intestinal aging and improve “leaky gut” in aging mice. Studies by Zhao et al (Zhao et al., 2022; Zhao et al., 2023). also show that Parishin intervention can ameliorate aging-induced cardiopulmonary fibrosis, alleviate aging-related gut microbiota dysbiosis, and inhibit vascular endothelial cell senescence by upregulating Klotho expression, thereby delaying vascular aging processes. In addition, the study by Liu et al (Liu et al., 2025). demonstrated that palescenoside can improve dextran sulfate sodium-induced colitis and anxiety-like behaviors by enhancing intestinal barrier function and modulating gut microbiota. The research by Ma et al (Ma et al., 2024). confirmed that palescenoside A exerts anti-oral squamous cell carcinoma effects by inhibiting the PI3K/AKT/mTOR signaling pathway and EMT process. The findings of Wang et al (Wang et al., 2021). showed that palescenoside C can provide neuroprotective effects in a rat middle cerebral artery occlusion model by suppressing oxidative stress and inflammatory responses. This study conducted untargeted metabolomic analysis on the tubers before bolting, tubers after bolting, and stems of three *Gastrodia elata* varieties. The results showed that the gastrodin abundance in the stems was significantly higher than in pre-bolting tubers (*P* < 0.05), which is consistent with the findings of Liu et al (Liu et al., 2015). Additionally, we found that the parishin C abundance in the stems of Hong Tianma and XueHong Tianma was extremely significantly higher than in pre-bolting tubers (*P* < 0.01), while the parishin C abundance in the stems of Wu Tianma was significantly higher than in pre-bolting tubers (*P* < 0.05). The one-way ANOVA results for overall flavonoids also indicated that the flavonoid abundance in the stems of all three *Gastrodia elata* varieties was extremely significantly higher than in pre-bolting tubers (*P* < 0.01). Since flavonoid content is closely related to antioxidant activity levels (Zhang et al., 2025), these results demonstrate that *Gastrodia elata* stems possess significant development value. Li et al (Li et al., 2024b; Li et al., 2024a). conducted chemical composition analysis on the stems and tubers of *Gastrodia elata* Bl. f. *elata* “Luema No. 1” and Wu Tianma using ultra-performance liquid chromatography-quadrupole time-of-flight mass spectrometry (UPLC-Q-TOF-MS/MS). The results showed that the stems contained more chemical components than the tubers, with flavonoids accumulating in the stems. These findings are consistent with the results of this study. It is noteworthy that while harvesting the medicinal tubers of *Gastrodia elata* annually, most of its stems are discarded or burned except for some used as raw materials for papermaking and biogas production (Jia et al., 2021), resulting in underutilization of *Gastrodia elata* stems. The findings of this study will potentially promote increased utilization of *Gastrodia elata* stems, provide theoretical basis for their rational development and utilization, and contribute to the advancement of the *Gastrodia elata* industry.

Additionally, this study identified two modules highly correlated with gastrodin and parishin C (*r* > 0.9). Among them, metabolites such as (R)-mandelic Acid and Isorhamnetin showed high correlation with gastrodin (*r* > 0.85), while Zeravschanoside and Austrobuxusin K exhibited high correlation with parishin C (*r* > 0.85). These metabolites may have certain relationships with the contents of gastrodin and parishin C, which provides a foundation for our next step to investigate the regulatory mechanisms associated with the accumulation of gastrodin and parishin C through transcriptome analysis. It should be noted that in this study, the sampling locations for pre-bolting and post-bolting tubers differed—pre-bolting tubers were sampled approximately 1 cm above the basal depression, while post-bolting tubers were sampled approximately 1 cm below the apical bud. This design was intended to track dynamic metabolic changes within the same plant to avoid individual variation, but it consequently introduced the potential confounding factor of tissue heterogeneity. The distribution of metabolites such as gastrodin and parishin C may inherently differ across anatomical regions of the tuber; therefore, the observed “increase after bolting” may partially reflect spatial location differences rather than a pure developmental transition. The conclusion regarding “increase after bolting” relies on the critical assumption that basal and apical tissues are metabolically comparable at baseline. Future studies should sample strictly from the same anatomical location across developmental stages, or conduct independent experiments to verify the spatial distribution patterns of metabolites, in order to more precisely disentangle the effects of developmental stage and tissue heterogeneity on metabolic changes.

### 4.2 Flavonoid biosynthesis and regulation during the bolting process of *Gastrodia elata*

Flavonoids, as important secondary metabolites in plants, are commonly found in flowers, leaves, and seeds, and play significant roles such as protecting plants from biotic stresses (herbivores, bacteria, fungi) and abiotic stresses (UV absorption) (Shen et al., 2022). The biosynthesis of flavonoids begins with the phenylalanine pathway, in which phenylalanine ammonia-lyase converts phenylalanine into cinnamic acid, followed by enzymatic reactions to form the basic structure of flavonoids (Zhang et al., 2025). In this study, the structures and properties of flavonoids (flavonoids, flavonols, anthocyanins, chalcones) during the bolting process of *Gastrodia elata* are diverse. During the bolting process of *Gastrodia elata*, the biosynthesis of flavonoids primarily involves flavonoid biosynthesis, anthocyanin biosynthesis, as well as the biosynthesis of flavones and flavonols, with these processes encompassing multiple interconnected metabolites. In terms of compound numbers, the majority of flavonoid compounds showed increased abundance in post-bolting tubers compared to pre-bolting tubers, and peaked in stems. This finding is inconsistent with the report that bolting reduces flavonoid biosynthesis and induces root lignification in *Angelica sinensis* (Li et al., 2022). This discrepancy may indicate fundamental differences in the bolting regulatory mechanisms between the orchid *Gastrodia elata* and the apiaceous plant *A. sinensis*. As a fully mycoheterotrophic plant, *G. elata* relies on *Armillaria* fungi for carbon and energy acquisition (Liu et al., 2024), and its reproductive strategy may differ significantly from that of autotrophic plants. It is noteworthy that our study found that the content of four anthocyanins increased in the tubers after bolting compared to those before bolting, while their levels decreased in the stems. Anthocyanins are important secondary metabolites in plants, mostly existing in glycoside forms, also known as anthocyanins (Hu et al., 2024). In addition to imparting various colors to plants, anthocyanins also play a regulatory role in plant development and environmental adaptation (Htay and Kil, 2021). Plant stress is defined as sudden changes in environmental conditions that adversely affect plant growth by interfering with various physiological, morphological, and metabolic processes (Rivero et al., 2021). Nutrient deficiency and pathogen attack are among the primary stress types for plants. Under stress conditions, plants undergo various metabolic and physiological changes, such as alterations in aboveground/underground biomass, reduction in photosynthesis and nutrient uptake, and inhibition of flowering and seed formation, thereby impairing plant growth. However, increased accumulation of anthocyanins can enhance plant tolerance to drought stress, salt stress, cold stress, nutrient stress, and UV radiation (Kaur et al., 2022b). In this study, the significantly higher content of four anthocyanins in tubers after bolting compared to before bolting may be attributed to the tuber’s need to provide nutrients for stem growth, resulting in nutritional deficiency in the tuber. This nutritional stress leads to substantial accumulation of anthocyanins in post-bolting tubers to scavenge reactive oxygen species (ROS) and protect plant tissues from stress damage. Similar results have also been observed in grapes, with studies showing that nitrogen and phosphorus deficiencies significantly promote the accumulation of anthocyanins in grape skin callus (Zheng et al., 2020). Recently, the study by Kaur et al (Kaur et al., 2022a). also demonstrated that phosphorus deficiency induces wheat to accumulate specific anthocyanins (including derivatives of cyanidin, delphinidin, and peonidin) in a genotype-dependent manner. In the stems of *Gastrodia elata*, the content of four anthocyanins decreased, which may indicate that anthocyanins are not the main cause of the color differences in the three cultivars of *Gastrodia elata* stems. As a fully mycoheterotrophic orchid, *Gastrodia elata* lacks chlorophyll and is incapable of photosynthesis (Liu et al., 2024); therefore, stem coloration is unrelated to chlorophyll. Flavonoids and other phenolic compounds are widely distributed in plant organs such as flowers, leaves, stems, and fruits, and are important contributors to tissue pigmentation (Cai et al., 2024; Qin et al., 2025), which may be associated with the color differences in *G. elata* stems. In this study, the overall flavonoid abundance in stems of all three varieties was significantly higher than in pre-bolting tubers (*P* < 0.01), supporting this inference. However, the specific causes of stem color variation among the three *G. elata* varieties remain to be fully elucidated, and further studies are needed to identify the key pigment components responsible for these color differences.

### 4.3 Biosynthesis and regulation of zeatin during the bolting process of *Gastrodia elata*

Zeatin belongs to the cytokinin (CKs) family and plays a crucial role in regulating cell growth and differentiation (Zhang et al., 2022). There are two known major biosynthetic pathways for cytokinins: the tRNA degradation pathway present in all organisms, and the *de novo* synthesis pathway identified in gymnosperms, angiosperms, and certain microorganisms. The tRNA degradation pathway primarily produces cis-isomers, while the *de novo* synthesis pathway forms trans-isomers (Perera et al., 2024). The metabolites detected in this study primarily involved cis-isomers. Cis-Zeatin O-glucoside and uridine 5’-diphosphate are two main products of cis-zeatin, which exhibited opposite patterns during the bolting process of *G. elata*: cis-Zeatin O-glucoside accumulated in the stems, while uridine 5’-diphosphate was consumed. Cytokinins can be classified into active forms, inactive precursors, or conjugated forms. Among them, free-base CKs, including isopentenyladenine (iP), trans-zeatin (tZ), cis-zeatin (cZ), and dihydrozeatin (DHZ), are considered to possess the highest biological activity. Common conjugated CKs mainly include glucoside-CKs, nucleoside-CKs, and nucleotide-CKs. Another less-studied group, methylthiolated CKs (2MeS-CKs), are derivatives of zeatin and iP, featuring a sulfhydryl group (-SH) at the 2-position of the adenine ring, which are generated through the tRNA degradation pathway (Gibb et al., 2020; Romanov and Schmülling, 2021; Perera et al., 2024). In this study, the differentially expressed metabolites in the zeatin biosynthesis pathway were predominantly conjugated forms, including glucosides, nucleosides, and methylthiolated compounds, with no direct involvement of the most bioactive free base types. The free base forms cis-zeatin and isopentenyladenine served as precursor substances for the differentially expressed metabolites cis-Zeatin O-glucoside, Uridine 5’-diphosphate, 5’-Methylthioadenosine, and S-Adenosylmethionine. Whether the bolting of *Gastrodia elata* involves the synthesis of free base-type metabolites and the mediation of differentially expressed genes warrants further exploration and validation through future transcriptome data analysis.

### 4.4 Plant hormone signal transduction during *Gastrodia elata* bolting

Jasmonic acid (JA) is an important class of lipid-derived plant hormones, initially identified as stress-related hormones in higher plants (Wang et al., 2020; Delfin et al., 2022). At least two JA biosynthesis pathways exist in plants: the octadecanoid pathway using linolenic acid (LA) as substrate, and the hexadecanoid pathway starting from hexadecatrienoic acid (HCA) (Luo et al., 2023). This study primarily focuses on the former pathway. Studies have shown that jasmonic acid and its amino acid-conjugated derivative (jasmonoyl-isoleucine: JA-Ile) are signaling molecules involved in regulating plant cell defense and development. Existing research has confirmed that JA directly participates in various physiological processes such as stamen development, senescence, and root growth, and can regulate the synthesis of multiple metabolites including phytoalexins and terpenoids (Gomi, 2021). Gibberellins regulate multiple growth and developmental processes in plants, including bolting and flowering (Jang et al., 2020; Chen et al., 2025). Previous studies have demonstrated that exogenous application of gibberellin can modulate stem elongation rate in wheat, while the application of chlormequat, a gibberellin biosynthesis inhibitor, can reduce flower stalk height during lettuce bolting (Wang et al., 2022). In land plants, the gibberellin biosynthesis pathway initiates in plastids, where two distinct diterpene cyclases convert farnesyl pyrophosphate (FPP) from precursors into ent-kaurene (Robil et al., 2025). Although approximately 136 forms of gibberellins have been identified in higher plants and fungi, only a few (GA1, GA3, GA4, and GA7) are biologically active, while the majority of gibberellins are either intermediates in the biosynthetic pathway or inactive forms (Jang et al., 2020). It has been reported that, through the hormone interaction network, jasmonic acid often synergizes with gibberellins to dynamically balance plant growth and defense processes (Liu and Timko, 2021). In this study, the abundance of JA in Wu Tianma reached its peak in the tubers after bolting, followed by a decrease in the stems. GA4 exhibited a consistent expression pattern with JA, both peaking in tuber abundance post-bolting while declining in the stems. For Wu Tianma, it is likely that JA and GA4 synergistically promoted the bolting process. Notably, we observed the synergistic expression pattern of JA and GA4 only in Wu Tianma. This variety-specific phenomenon may reflect genetic differences in the bolting regulatory mechanisms among different *G. elata* varieties. As one of the most widely cultivated varieties, Wu Tianma may possess a distinct hormone response network compared to other varieties. Additionally, differences in symbiotic efficiency or interaction patterns with *Armillaria* fungi among varieties might affect the activation levels of hormone signals. Future studies employing comparative transcriptomics or hormone treatment experiments could further elucidate the variety-specific molecular mechanisms regulating bolting in *G. elata*. However, it should be noted that the sampling method employed in this study may introduce a potential confounding factor. Since pre-bolting and post-bolting tuber samples were collected sequentially from the same individual plants, the initial wounding caused by sampling could have activated plant defense responses, including the jasmonic acid signaling pathway. Considering that JA is a well-established wound-responsive hormone (Wang et al., 2020; Nie et al., 2024), the elevated JA abundance observed in post-bolting tubers of Wu Tianma may partially reflect a wounding-induced response rather than solely a developmental transition. Therefore, while our findings suggest a synergistic role of JA and GA4 in bolting, the potential influence of wounding stress cannot be entirely excluded. Future studies should adopt independent sampling strategies—such as using different plants for each time point or including unwounded controls—to more precisely delineate the effects of bolting from those of wounding stress on metabolic reprogramming.

### 4.5 Changes in energy metabolites during the bolting process of *Gastrodia elata* and their biological significance

In this study, we observed significant decreases in energy metabolites such as sucrose, UDP-GlcNAc, and Fru-6P in *Gastrodia elata* tubers after bolting (**Figure 8**). As a non-photosynthetic mycoheterotrophic plant, *G. elata* relies entirely on *Armillaria* spp. fungi colonizing its tubers for carbon and energy acquisition (Zeng et al., 2018). Previous studies have shown that sucrose is the most abundant sugar in *G. elata* tubers at all developmental stages and likely represents the primary form of carbon transported at the fungus-orchid interface (Ho et al., 2020). The sucrose transporter GeSUT4, which is highly expressed in young tubers and localized to both the plasma membrane and tonoplast of symbiotic cells, participates in active sucrose uptake and intracellular allocation (Ho et al., 2020). Additionally, genes involved in starch and sucrose metabolism are expressed in *G. elata* vegetative tubers (Tsai et al., 2016), indicating active carbon metabolic activity in the tubers.

The rapid elongation of flowering stems and subsequent reproductive processes require substantial energy and carbon skeletons, likely inducing the catabolic mobilization of stored starch and sucrose in tubers to support reproductive growth. This energy reallocation process is particularly critical in the *G. elata*-fungus symbiotic system—fungal-derived carbon is transported to tubers in the form of sucrose (Ho et al., 2020) and subsequently remobilized to reproductive organs during bolting. Therefore, the decrease in energy metabolites after bolting primarily reflects the mobilization of tuber reserves for reproductive growth rather than changes in photosynthetic input. This process may be regulated by sucrose transporters such as GeSUT4 (Ho et al., 2020) and is closely associated with dynamic changes in the *G. elata*-*Armillaria* symbiotic relationship (Liu et al., 2024).

### 4.6 Applicability and limitations of indoor cultivation conditions

Indoor cultivation was chosen to standardize environmental conditions and reduce external interference with metabolite analysis, thereby enabling a more accurate assessment of the effects of developmental stages and variety factors on the metabolic characteristics of *Gastrodia elata*. It should be noted that under natural conditions, *G. elata* typically grows in understory and similarly shaded habitats (Chen et al., 2019). Indoor cultivation conditions (such as light intensity and diurnal temperature variation) differ to some extent from the natural environment, which may influence the absolute contents of bioactive compounds.

However, the primary objective of this study was to compare the relative metabolic differences among three *G. elata* varieties across three key developmental stages (pre-bolting tubers, post-bolting tubers, and stems), rather than to determine absolute contents of active ingredients. Under the same indoor cultivation conditions, all samples were subjected to identical environmental treatments, ensuring that environmental factors exerted consistent and uniform effects across all treatment groups. Therefore, the metabolic differences among varieties and developmental stages are primarily determined by genetic background and developmental programs, and indoor cultivation conditions do not alter the significance of these relative differences.

Accordingly, the main conclusions of this study are reliable in terms of relative comparisons. Future research may further combine field experiments to validate the generalizability of the metabolic changes observed under indoor cultivation to natural growth conditions.

## 5 Conclusion

This study comprehensively investigated the dynamic changes of metabolites in three key developmental stages of *Gastrodia elata* (pre-bolting tubers, post-bolting tubers, and stems) through metabolomic analysis. The results showed that the two main types of compounds affecting the developmental stages of *Gastrodia elata* were lipids and flavonoids. The abundance of flavonoids, coumarins, lignans, and organic nitrogen compounds peaked in the stems, while the abundance of lipids, organic acids, amino acids, and carbohydrates reached their peak in the tubers after bolting. We identified 43 potential signature metabolites that may distinguish the three cultivars of *Gastrodia elata*. We also found that flavonoid biosynthesis, zeatin metabolism, and plant hormone signal transduction are the core metabolic pathways regulating the growth and development of *Gastrodia elata*. The core energy metabolism network during the three developmental stages of *Gastrodia elata* consists of amino sugar and nucleotide sugar metabolism, starch and sucrose metabolism, glycolysis/gluconeogenesis, and pentose and glucuronate interconversions. We identified JA as a metabolite potentially associated with the bolting process in Wu Tianma. Additionally, the abundances of gastrodin and parishin C in the stems of three *Gastrodia elata* cultivars were significantly higher than those in pre-bolting tubers (*P* < 0.05). Our findings will provide a theoretical foundation for gaining deeper insights into the critical developmental stages of *Gastrodia elata* and the rational exploitation of its stem resources.

## Data Availability

The original contributions presented in the study are included in the article/**Supplementary Material**. Further inquiries can be directed to the corresponding author.

## References

[B1] Berentsen R. Benlloch R. Visser P. Madueño F. Balanzà V. (2024). A reduced vernalization requirement is a key component of the early-bolting trait in globe artichoke (Cynara cardunculus var. scolymus). iScience 27, 110829. doi: 10.1016/j.isci.2024.110829. PMID: 39297164 PMC11409005

[B2] Cai O. Zhao W. Q. Kong S. X. Jiang S. J. Yao W. J. Li L. . (2024). Mechanism regulation of culm color formation in plants. Mol. Plant Breed., 1–8.

[B3] Chen C. Q. (2024). Research on the development of Gastrodia elata industry chain in Xiaocaoba Town, Yiliang County. Yunnan Agric. Univ. doi: 10.27458/d.cnki.gynyu.2024.000761

[B4] Chen H. Y. Qi Y. T. Wang Y. Liu J. Lu R. R. Zhao X. H. . (2025). LsBLH2–LsOFP6–LsKANT3 module regulates bolting by orchestrating the gibberellin biosynthesis and metabolism in lettuce. Plant Biotechnol. J. 23, 1668–1682. doi: 10.1111/pbi.14614. PMID: 39932895 PMC12018825

[B5] Chen L. Wang Y. C. Qin L. Y. He H. Y. Yu X. L. Yang M. Z. . (2019). Dynamics of fungal communities during Gastrodia elata growth. BMC Microbiol. 19, 1–11. doi: 10.1186/s12866-019-1501-z. PMID: 31291888 PMC6617676

[B6] Cheng L. J. Deng Y. (2021). Characterization by HPLC of p-Hydroxybenzyl alcohol biotransformation to gastrodin *in vivo*. Nat. Prod. Commun. 16, 9. doi: 10.1177/1934578X211035069. PMID: 41930703

[B7] Delfin J. C. Kanno Y. Seo M. Kitaoka N. Matsuura H. Tohge T. . (2022). AtGH3. 10 is another jasmonic acid-amido synthetase in Arabidopsis thaliana. Plant J. 110, 1082–1096. doi: 10.1111/tpj.15724. PMID: 35247019

[B8] Ding L. Zhou T. Wu T. S. Yang C. G. Li X. Xiong H. X. . (2025). Status and suggestions of changes of producing area and industrial development of Gastrodia elata. J. Chin. Med Mater 48, 1–7. doi: 10.13863/j.issn1001-4454.2025.01.001

[B9] Du H. L. Liu C. Mei C. Zhan J. F. (2025). Progress in research on flavor compounds in Gastrodia elata. J. Food. Bioact 29, 29–37. doi: 10.26599/JFB.2025.95029404. PMID: 41311685

[B10] Fan S. C. Liu D. X. Chen C. (2021). Analysis on the development trend and hot spots of Gastrodia elata Bl. research based on bibliometrics. Chin. J. Mod Appl. Pharm. 38, 2754–2762. doi: 10.13748/j.cnki.issn1007-7693.2021.21.020

[B11] Fu A. Z. Bai C. M. Wang M. Fernie A. R. Wang H. W. Jing Z. X. . (2025). Key metabolic pathways across sponge gourd (Luffa aEgyptiaca Mill.) development and ripening. Hortic. Plant J. doi: 10.1016/j.hpj.2025.07.011. PMID: 41936479

[B12] Gibb M. Kisiala A. B. Morrison E. N. Emery R. J. N. (2020). The origins and roles of methylthiolated cytokinins: evidence from among life kingdoms. Front. Cell Dev. Biol. 8. doi: 10.3389/fcell.2020.605672. PMID: 33240900 PMC7680852

[B13] Gomi K. (2021). Jasmonic acid pathway in plants 2.0. Int. J. Mol. Sci. 22, 3506. doi: 10.3390/ijms22073506. PMID: 33805251 PMC8036587

[B14] Gong C. X. Ma C. Irge D. D. Li S. M. Chen S. M. Zhou S. X. . (2023). Gastrodia elata and parishin ameliorate aging induced ‘leaky gut’ in mice: correlation with gut microbiota. Biomed. J. 46, 100547. doi: 10.1016/j.bj.2022.07.001. PMID: 35811058 PMC10345228

[B15] Guo J. X. Xie J. Jiang L. S. Gao J. H. Rao C. L. Zuo L. L. (2022). Analysis on development status of Gastrodiae Rhizoma health food. Chin. Trad Herbal Drugs 53, 2247–2254. doi: 10.7501/j.issn.0253-2670.2022.07.034

[B16] Ho L. H. Lee Y. I. Hsieh S. Y. Lin I. S. Wu Y. C. Ko H. Y. . (2020). GeSUT4 mediates sucrose import at the symbiotic interface for carbon allocation of heterotrophic Gastrodia elata (Orchidaceae). Plant Cell Environ. 44, 20–33. doi: 10.1111/pce.13833. PMID: 32583877

[B17] Htay N. A. Kil K. C. (2021). Abiotic stress-induced anthocyanins in plants: their role in tolerance to abiotic stresses. Physiol. Plant 172, 1711–1723. doi: 10.1111/ppl.13373. PMID: 33605458

[B18] Hu Y. R. Huang X. L. Ye J. W. (2024). Research progress of anthocyanin synthesis and regulatory factors in plants. Life. Sci. Res. 28, 493–502. doi: 10.16605/j.cnki.1007-7847.2024.11.0196

[B19] Hu Y. P. Li S. N. Xu J. Zhou T. Jiang W. K. Ou X. H. (2025). Identification of five ecotypes of Gastrodia elata based on chloroplast genome SSR molecular marker. Chin. Trad Herbal Drugs 56, 1747–1754. doi: 10.7501/j.issn.0253-2670.2025.05.023

[B20] Jang G. Yoon Y. Choi Y. D. (2020). Crosstalk with jasmonic acid integrates multiple responses in plant development. Int. J. Mol. Sci. 21, 305. doi: 10.3390/ijms21010305. PMID: 31906415 PMC6981462

[B21] Jia C. Q. Ma R. Su W. C. Gui G. B. Yu J. S. (2021). Cultivation of Pleurotus ostreatus with stem waste of Gastrodia elata and analysis of its nutrient components. Food. Industr 42, 338–343.

[B22] Jiang X. Ran D. D. Wang X. W. Zhang X. B. Ou X. H. Pan J. . (2026). Identification and analysis of bHLH genes related to color formation of Gastrodia elata stem. Chin. J. Exp. Trad Med. Formulae 32, 202–209. doi: 10.13422/j.cnki.syfjx.20260117

[B23] Jin Y. Y. (2024). Fine mapping and gene function verification of key QTL loci qBT2 and qBT7.2 for bolting and flowering in radish. Guizhou Univ. doi: 10.27047/d.cnki.ggudu.2024.003438

[B24] Kaur S. Kumari A. Sharma N. Pandey A. K. Garg M. (2022a). Physiological and molecular response of colored wheat seedlings against phosphate deficiency is linked to accumulation of distinct anthocyanins. Plant Physiol. Biochem. 170, 338–349. doi: 10.1016/j.plaphy.2021.12.017. PMID: 34959054

[B25] Kaur S. Tiwari V. Kumari A. Chaudhary E. Sharma A. Ali U. . (2022b). Protective and defensive role of anthocyanins under plant abiotic and biotic stresses: an emerging application in sustainable agriculture. J. Biotechnol. 361, 12–29. doi: 10.1016/j.jbiotec.2022.11.009. PMID: 36414125

[B26] Kim D. W. Kim J. K. Gebru Y. A. Kim Y. H. Choi H. S. Kim M. K. (2022). Identification of novel parishin compounds from the twig of Maclura tricuspidata and comparative analysis of parishin derivatives in different parts. Molecules 28, 7. doi: 10.3390/MOLECULES28010007. PMID: 36615203 PMC9822251

[B27] Li B. J. Bai J. Q. He W. W. Wang X. P. Zhang W. Yang Q. (2024a). The chemical constituents of stems and tubers of G.elata were analyzed based on UPLC-Q-TOF-MS / MS technology. Mod Chin. Med. 44, 99–105. doi: 10.13424/j.cnki.mtcm.2024.03.019

[B28] Li M. L. Cui X. W. Jin L. Li M. F. Wei J. H. (2022). Bolting reduces ferulic acid and flavonoid biosynthesis and induces root lignification in Angelica sinensis. Plant Physiol. Biochem. 170, 171–179. doi: 10.1016/j.plaphy.2021.12.005. PMID: 34891073

[B29] Li N. Cui X. L. Lu Y. J. Zhang F. Guo S. Su Y. . (2025). Spatial distribution analysis of secondary metabolites in bolted and unbolted Saposhnikovia divaricata by atmospheric pressure laser ablation carbon fiber ionization mass spectrometry imaging. Anal. Chim. Acta 1358, 344096. doi: 10.1016/j.aca.2025.344096. PMID: 40374248

[B30] Li B. J. He W. W. Bai J. Q. Wang X. P. Li J. M. Li X. D. (2024b). Analysis of chemical components in stem and tuber of Gastrodia elata Bl.f.elata”Luema No.1” by UPLC-Q-TOF-MS/MS. Mod Chin. Med. 26, 788–798. doi: 10.13313/j.issn.1673-4890.20240111002

[B31] Li Y. Liu X. Q. Liu S. S. Liu D. H. Wang X. Wang Z. M. (2019). Transformation mechanisms of chemical ingredients in steaming process of Gastrodia elata Blume. Molecules 24, 3159. doi: 10.3390/molecules24173159. PMID: 31480235 PMC6749462

[B32] Li J. Liu X. X. Luo M. M. Huang T. M. Xie D. D. Zhang H. Y. . (2022). Effect of seedlings frozen storage on early bolting, biomass and quality of Angelica sinensis. J. Gansu Agric. Univ. 57, 90–97. doi: 10.13432/j.cnki.jgsau.2022.02.011

[B33] Li H. Qian R. Tian N. Li Y. H. Jiang C. Yuan Y. . (2020). Identification of Gastrodia elata and its hybrid by polymerase chain reaction. China J. Chin. Mater Med. 45, 3666–3671. doi: 10.19540/j.cnki.cjcmm.20200527.102. PMID: 32893556

[B34] Li N. Wang K. J. Chen J. J. Zhou J. (2007). Phenolic compounds from the rhizomes of Gastrodia elata. J. Asian Nat. Prod. Res. 9, 373–377. doi: 10.1080/10286020600780979. PMID: 17613623

[B35] Liu Y. Gao J. L. Peng M. Meng H. Y. Ma H. B. Cai P. P. . (2018). A review on central nervous system effects of gastrodin. Front. Pharmacol. 9. doi: 10.3389/fphar.2018.00024. PMID: 29456504 PMC5801292

[B36] Liu H. Timko M. P. (2021). Jasmonic acid signaling and molecular crosstalk with other phytohormones. Int. J. Mol. Sci. 22, 2914. doi: 10.3390/ijms22062914. PMID: 33805647 PMC8000993

[B37] Liu F. Yan H. Y. Xu Y. X. Liu Y. M. Liu D. H. (2023). Study on the planting and warming time in hybrid seed production of Gastrodia elata f. glauca and Gastrodia elata f. elata. Biotic Resour. 45, 177–184. doi: 10.14188/j.ajsh.2023.02.009

[B38] Liu J. J. Yang X. Q. Li Z. Y. Miao J. Y. Li S. B. Zhang W. P. . (2024). The role of symbiotic fungi in the life cycle of Gastrodia elata Blume (Orchidaceae): a comprehensive review. Front. Plant Sci. 14. doi: 10.3389/FPLS.2023.1309038. PMID: 38264031 PMC10804856

[B39] Liu J. Y. Zhao H. Wang Y. F. Zhou C. X. Zhang Y. Y. Liu M. (2015). Comparative study on chemical constituents in different parts of Wumei Gastrodia elata Blume and wild Gastrodia elata Blume. Spec Wild Econ Anim. Plant Res. 37, 1001–4721. doi: 10.16720/j.cnki.tcyj.2015.02.014

[B40] Liu X. X. Zhou H. J. Yang N. Yang L. J. Zi Z. Y. Han Y. L. . (2025). Parishin from Gastrodia elata ameliorates DSS induced colitis and anxiety-like behavior in mice by regulating intestinal barrier function and microviota-gut-brain axis. Phytomedicine 145, 157019. doi: 10.1016/j.phymed.2025.157019. PMID: 40582206

[B41] Luo X. P. Tu M. X. Zhou H. D. Li Z. G. (2023). Novel role of jasmonic acid signaling in plant response to environmental stress. J. Yunnan Norm Univ: Natural Sci. Ed 43, 1–8. doi: 10.7699/j.ynnu.ns-2023-045

[B42] Ma L. Liu Z. B. Kim E. Y. Huang K. Kim C. Y. Kim H. J. . (2024). Parishin A inhibits oral squamous cell carcinoma via the AKT/mTOR signaling pathway. Pharmaceuticals 17, 1277. doi: 10.3390/ph17101277. PMID: 39458918 PMC11510427

[B43] Nie R. R. Chen D. Hu T. T. Zhang S. Y. Qu G. Q. (2024). A review: the role of jasmonic acid in tomato flower and fruit development. Plant Mol. Biol. Rep. 43, 1–10. doi: 10.1007/S11105-024-01505-X. PMID: 41933263

[B44] Perera I. Kisiala A. Thompson K. A. Emery R. J. N. (2024). Soil health improvements under cover crops are associated with enhanced soil content of cytokinins. Plant Biol. 27, 265–278. doi: 10.1111/plb.13743. PMID: 39642005 PMC11846634

[B45] Qin S. S. Liang Y. Xie Y. Y. Wei G. L. Lin Q. Qin W. Q. . (2025). Genome-wide analysis of the bHLH gene family in Spatholobus suberectus identifies SsbHLH112 as a regulator of flavonoid biosynthesis. BMC Plant Biol. 25, 594. doi: 10.1186/S12870-025-06452-7. PMID: 40329176 PMC12054232

[B46] Qin S. H. Wu S. Y. Yang L. Xu F. Q. Hu J. M. (2023). Chemical composition of flower stem of Gastrodia elata. Chin. Trad Herbal Drugs 54, 7717–7722. doi: 10.7501/j.issn.0253-2670.2023.23.013

[B47] Rivero R. M. Mittler R. Blumwald E. Zandalinas S. I. (2021). Developing climate-resilient crops: improving plant tolerance to stress combination. Plant J. 109, 373–389. doi: 10.1111/tpj.15483. PMID: 34482588

[B48] Robil J. M. Awale P. McSteen P. Best N. B. (2025). Gibberellins: extending the green revolution. J. Exp. Bot. 76, 1837–1853. doi: 10.1093/jxb/erae476. PMID: 39570614 PMC12066124

[B49] Romanov G. A. Schmülling T. (2021). On the biological activity of cytokinin free bases and their ribosides. Planta 255, 27. doi: 10.1007/s00425-021-03810-1. PMID: 34940934 PMC8702413

[B50] Shen T. Liu H. G. Wang Y. Z. (2023). Herbal textual and key problems discussion in modern resource utilization of Gastrodiae Rhizoma. Chin. Trad Herbal Drugs 54, 6106–6117. doi: 10.7501/j.issn.0253-2670.2023.18.028

[B51] Shen N. Wang T. F. Gan Q. Liu S. Wang L. Jin B. (2022). Plant flavonoids: classification, distribution, biosynthesis, and antioxidant activity. Food Chem. 383, 132531. doi: 10.1016/j.foodchem.2022.132531. PMID: 35413752

[B52] Shi Z. L. Ma Z. L. Wang Y. Li D. Guo Y. F. Xu L. P. . (2026). Symbiotic cultivation of Gastrodia elata: Armillaria strain selection reprograms carbon allocation to balance tuber yield and phenolic glycosides. Horticulturae 12, 181. doi: 10.3390/HORTICULTURAE12020181. PMID: 41725453

[B53] Tsai C. C. Wu K. M. Chiang T. Y. Huang C. Y. Chou C. H. Li S. J. . (2016). Comparative transcriptome analysis of Gastrodia elata (Orchidaceae) in response to fungus symbiosis to identify gastrodin biosynthesis-related genes. BMC Genomics 17, 212. doi: 10.1186/s12864-016-2508-6. PMID: 26960548 PMC4784368

[B54] Wang D. C. (2024). Effects of different temperatures and storage periods on the growth and material metabolism of apical buds of Gastrodia elata. Guizhou Univ. doi: 10.27047/d.cnki.ggudu.2024.000871

[B55] Wang Y. L. Bai M. T. Wang X. Peng Z. L. Cai C. Y. Xi J. J. . (2024). Gastrodin: a comprehensive pharmacological review. Naunyn-Schmiedeberg’s Arch. Pharmacol. 397, 3781–3802. doi: 10.1007/s00210-023-02920-9. PMID: 38165423

[B56] Wang T. Chen H. B. Xia S. Y. Chen X. F. Sun H. Xu Z. X. (2021). Ameliorative effect of parishin C against cerebral ischemia-induced brain tissue injury by reducing oxidative stress and inflammatory responses in rat model. Neuropsychiatr. Dis. Treat. 17, 1811–1823. doi: 10.2147/ndt.s309065. PMID: 34113111 PMC8187103

[B57] Wang J. Q. Li D. L. Chen N. Chen J. J. Mu C. J. Yin K. . (2020). Plant grafting relieves asymmetry of jasmonic acid response induced by wounding between scion and rootstock in tomato hypocotyl. PloS One 15, e0241317. doi: 10.1371/JOURNAL.PONE.0241317. PMID: 33232332 PMC7685457

[B58] Wang D. Liu W. Lu M. J. Xu Q. (2025). Neuropharmacological effects of Gastrodia elata Blume and its active ingredients. Front. Neurol. 16. doi: 10.3389/FNEUR.2025.1574277. PMID: 40371076 PMC12074926

[B59] Wang D. C. Liu J. D. Tang L. S. Yang Y. Huang N. Huang M. J. . (2024a). Study on differential metabolites of Gastrodia elata tuber before and after bolting based on non-targeted metabolomics. Seed 43, 97–104+26. doi: 10.16590/j.cnki.1001-4705.2024.07.097

[B60] Wang S. L. Luo C. Sun L. Ning K. Chen Z. J. Yang J. J. . (2022). LsRGL1 controls the bolting and flowering times of lettuce by modulating the gibberellin pathway. Plant Sci. 316, 111175. doi: 10.1016/j.plantsci.2021.111175. PMID: 35151458

[B61] Wang J. Song L. Gong X. Xu J. F. Li M. H. (2020). Functions of jasmonic acid in plant regulation and response to abiotic stress. Int. J. Mol. Sci. 21, 1446. doi: 10.3390/ijms21041446. PMID: 32093336 PMC7073113

[B62] Wang Q. Wang T. Xu D. Wang Z. D. Huang J. Li Y. . (2023). Research progress of Gastrodia elata. Sichuan Agric. Sci. Technol. 12, 103–106. doi: 10.3969/j.issn.1004-1028.2023.12.029. PMID: 35900448

[B63] Wang L. Xiao H. B. Liang X. M. Wei L. X. (2007). Identification of phenolics and nucleoside derivatives in Gastrodia elata by HPLC-UV-MS. J. Sep Sci. 30, 1488–1495. doi: 10.1002/jssc.200600469. PMID: 17623430

[B64] Wang S. M. Yang S. Q. Hu Z. F. Liu J. J. Peng J. H. Ran Q. Y. . (2024). Effects of different light qualities on the bolting characteristics of Gastrodia elata f.glauca. Chin. Wild Plant Resour. 43, 57–64. doi: 10.3969/j.issn.1006-9690.2024.11.008. PMID: 35900448

[B65] Wang D. C. Yang Y. Huang N. Huang M. J. Guo G. Shen S. M. (2024b). Chemical constituents from the fresh tuber of Gastrodia elata with dark red pedicel and their antibacterial activities. J. Chin. Med Mater 47, 2768–2773. doi: 10.13863/j.issn1001-4454.2024.11.017

[B66] Wang X. Yang W. Z. Lv J. T. Liao X. Y. (2024). Study on the uptake of gastrodin in the liver. Heliyon 10, e36031. doi: 10.1016/j.heliyon.2024.e36031. PMID: 39229547 PMC11369432

[B67] Xiao G. R. Tang R. Yang N. Chen Y. H. (2023). Review on pharmacological effects of gastrodin. Arch. Pharmacal Res. 46, 744–770. doi: 10.1007/s12272-023-01463-0. PMID: 37749449

[B68] Yuan Q. S. Wang H. Jiang W. K. Ou X. H. Xu J. Wang X. A. . (2020). Dissection of seed development of Gastrodia elata at different temperatures. China J. Chin. Mater Med. 45, 485–490. doi: 10.19540/j.cnki.cjcmm.20191204.109. PMID: 32237504

[B69] Zeng X. Li J. X. Chen T. Y. Li Y. Y. Guo S. X. (2023). Global metabolic profile and multiple phytometabolites in the different varieties of Gastrodia elata Blume. Front. Plant Sci. 14. doi: 10.3389/FPLS.2023.1249456. PMID: 37915510 PMC10616830

[B70] Zeng X. Li Y. Y. Ling H. Chen J. Guo S. X. (2018). Revealing proteins associated with symbiotic germination of Gastrodia elata by proteomic analysis. Bot. Stud. 59, 8. doi: 10.1186/s40529-018-0224-z. PMID: 29511914 PMC5840113

[B71] Zhan H. D. Zhou H. Y. Sui Y. P. Du X. L. Wang W. H. Dai L. . (2016). The rhizome of Gastrodia elata Blume - an ethnopharmacological review. J. Ethnopharmacol. 189, 361–385. doi: 10.1016/j.jep.2016.06.057. PMID: 27377337

[B72] Zhang Y. Cai J. R. Lu W. Xu S. J. Qu M. D. Zhao S. . (2022). Comprehensive network-based analyses reveal novel renal function-related targets in acute kidney injury. Front. Genet. 13. doi: 10.3389/fgene.2022.907145. PMID: 35860471 PMC9289212

[B73] Zhang Y. J. Hou L. J. Hu J. Wang X. C. Guo S. J. Xie H. Q. . (2025). American ginseng fruit: Antioxidant capacity, bioactive components, and biosynthesis mechanism during development. Food Res. Int. 203, 115884. doi: 10.1016/j.foodres.2025.115884. PMID: 40022396

[B74] Zhang Y. S. Long Y. Zheng Z. P. Zhang H. M. Liu X. H. Cao D. . (2022). Cloning and expression of genes related to zeatin synthesis pathway. Seed 41, 55–60. doi: 10.16590/j.cnki.1001-4705.2022.08.055

[B75] Zhang C. Q. Zhang X. G. Cheng F. X. He Y. J. Mi J. (2011). Quality characteristic of Sichuan Gastrodia elata tuber at different sexual developmental stages. J. Anhui Agric. Sci. 39, 15936–15938. doi: 10.13989/j.cnki.0517-6611.2011.26.185

[B76] Zhao X. X. Zhou S. X. Liu Y. Gong C. X. Xiang L. Li S. M. . (2023). Parishin alleviates vascular ageing in mice by upregulation of Klotho. J. Cell. Mol. Med. 27, 1398–1409. doi: 10.1111/jcmm.17740. PMID: 37032511 PMC10183705

[B77] Zhao X. X. Zhou S. X. Yan R. Gong C. X. Gui Q. F. Zhang Q. . (2022). Parishin from Gastrodia elata ameliorates aging phenotype in mice in a gut microbiota-related manner. Front. Microbiol. 13. doi: 10.3389/fmicb.2022.877099. PMID: 35547139 PMC9083111

[B78] Zheng H. Z. Wei H. Guo S. H. Yang X. Feng M. X. Jin X. Q. . (2020). Nitrogen and phosphorus co-starvation inhibits anthocyanin synthesis in the callus of grape berry skin. J. Plant Biotechnol. 142, 313–325. doi: 10.1007/s11240-020-01864-9. PMID: 41933263

[B79] Zhu J. F. Liu B. Xu Q. Meng Z. Y. Dai X. Y. Mai Y. Q. . (2024). Dynamic change patterns of seven active components of Tianma (Gastrodia elata) at different developmental stages and confirmation of quality difference markers. Chin. Arch. Trad Chin. Med. 42, 197–203+293.

[B80] Zhu H. Y. Pei Z. X. Zhang L. Dong J. H. Ji P. Z. (2025). Non-targeted metabolomics-mediated elucidation of metabolite changes in Polygonatum kingianum during traditional steaming with black beans. Front. Nutr. 12. doi: 10.3389/fnut.2025.1581459. PMID: 40290657 PMC12021634

