# Supplementary material for: Unraveling the metabolic network and key bioactive compound accumulation during critical developmental stages of Gastrodia elata
Source: Front Plant Sci. 2026 May 1;17:1804747. doi: 10.3389/fpls.2026.1804747 (PMC13176193; doi:10.3389/fpls.2026.1804747)
Supplement: Supplementary file 1 [file DataSheet1.docx]

Supplementary Material

1. **Supplementary Tables**

**Table S1.** PERMANOVA results showing the effects of developmental stage and variety on the metabolomic profiles of *Gastrodia elata* (based on Bray-Curtis distance, 999 permutations).

| Factor | Df | SumOfSqs | R^2^ | F | Pr(>F) | Significance |
| --- | --- | --- | --- | --- | --- | --- |
| Stage | 2 | 1.0830 | 0.6346 | 31.3158 | 0.001 | *** |
| Variety | 2 | 0.2432 | 0.1425 | 7.0324 | 0.001 | *** |

Notes: Df: degrees of freedom; SumOfSqs: sum of squares; R²: proportion of variance explained by each factor; F: F-statistic; Pr(>F): p-value based on permutation test. Significance levels: ****P* < 0.001.

**Table S2. Statistical analysis of** Gastrodin **and** Parishin C **levels following data normalization using one-way ANOVA.**

|  | T-a-H | T-a-W | T-a-XH | T-b-H | T-b-W | T-b-XH | S-H | S-W | S-XH |
| --- | --- | --- | --- | --- | --- | --- | --- | --- | --- |
| Parishin C | 15.23±0.18^Bb^ | 10.84±0.80^Bc^ | 7.23±0.00^Cc^ | 21.63±0.33^Aa^ | 22.65±0.05^Aa^ | 23.17±0.05^Aa^ | 20.82±1.63^Aa^ | 16.21±2.64^Bb^ | 17.29±0.07^Bb^ |
| Gastrodin | 18.45±0.10^Cc^ | 15.10±0.51^Ab^ | 13.15±0.18^Bb^ | 21.28±0.19^Bb^ | 20.55±0.12^Aa^ | 21.05±0.25^Aa^ | 23.40±0.79^Aa^ | 18.82±3.29^Aab^ | 21.00±0.13^Aa^ |

Notes: different capital letters in each column indicate significance *P* < 0.01, and different lowercase letters indicate *P* < 0.05. Data are expressed as the mean ± standard deviation (n = 3).

**Table S3.** Trait data used for WGCNA


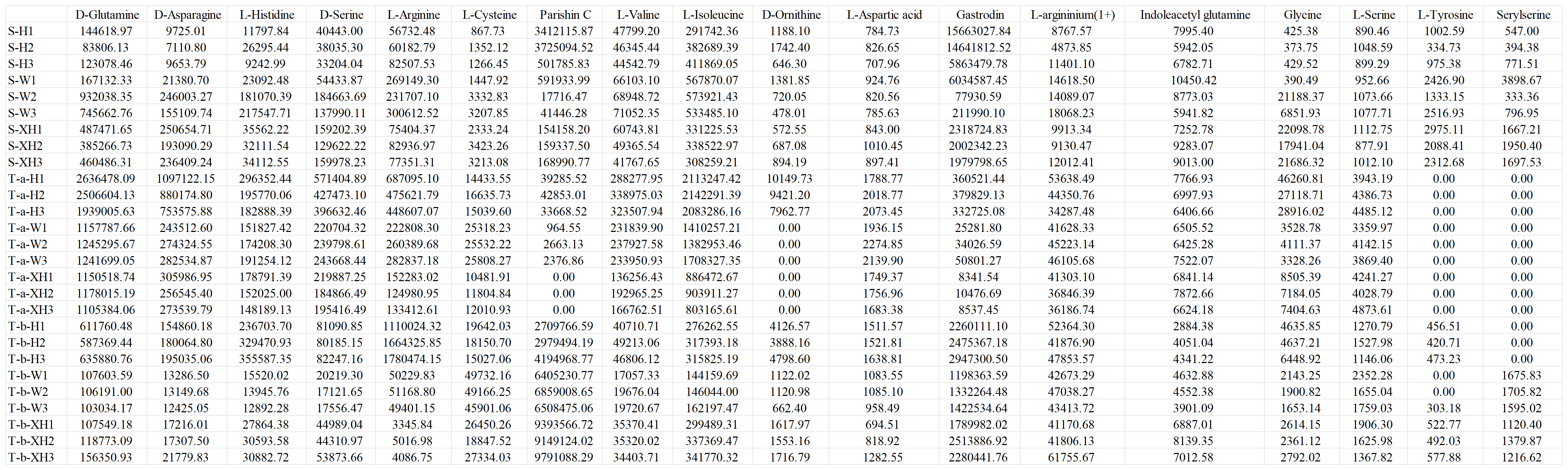


**Table S4. Overall one-way ANOVA results of principal compound categories across three *Gastrodia elata* varieties at distinct developmental stages.**

|  | T-a-H | T-a-W | T-a-XH | T-b-H | T-b-W | T-b-XH | S-H | S-W | S-XH |
| --- | --- | --- | --- | --- | --- | --- | --- | --- | --- |
| Flavonoids and derivatives | 8.76±0.03^Cc^ | 8.48±0.04^Bb^ | 8.04±0.02^Cc^ | 10.80±0.18^Bb^ | 10.39±0.19^Aa^ | 10.95±0.03^Bb^ | 11.48±0.17^Aa^ | 10.82±0.40^Aa^ | 11.13±0.03^Aa^ |
| Lipids and lipid-like molecules | 10.10±0.03^Bb^ | 10.04±0.04^Bb^ | 9.73±0.06^Cc^ | 11.15±0.14^Aa^ | 11.59±0.09^Aa^ | 11.46±0.07^Aa^ | 9.93±0.14^Bb^ | 10.26±0.28^Bb^ | 10.15±0.04^Bb^ |
| Amino acids, peptides, and analogues | 9.75±0.04^Bc^ | 9.49±0.03^Cc^ | 9.36±0.03^Cc^ | 11.77±0.10^Aa^ | 11.74±0.08^Aa^ | 11.05±0.08^Aa^ | 10.32±0.32^Bb^ | 10.64±0.37^Bb^ | 10.80±0.01^Bb^ |
| Carbohydrates and carbohydrate conjugates | 18.38±0.07^Cc^ | 9.92±0.06^Bb^ | 9.51±0.05^Cc^ | 11.95±0.07Aa | 11.24±0.21^Aa^ | 11.73±0.05^Aa^ | 11.40±0.15^Bb^ | 10.85±0.26^Aa^ | 11.00±0.01^Bb^ |
| Coumarins, Isocoumarins and derivatives | 8.58±0.07^Bb^ | 8.34±0.04^Bb^ | 8.05±0.05^Cc^ | 10.15±0.18^Aa^ | 10.00±0.15^Aa^ | 10.48±0.01^Bb^ | 10.52±0.23^Aa^ | 10.29±0.27^Aa^ | 10.62±0.03^Aa^ |
| Organic acids and derivatives | 9.31±0.02^Cc^ | 9.22±0.01^Cc^ | 9.28±0.04^Cc^ | 10.63±0.09^Aa^ | 11.65±0.06^Aa^ | 10.95±0.04^Aa^ | 10.03±0.22^Bb^ | 10.07±0.04^Bb^ | 10.29±0.04Bb |
| Cinnamaldehydes, Cinnamic acids and derivatives | 9.78±0.04^Bb^ | 9.27±0.14^Bb^ | 8.69±0.04^Cc^ | 12.11±0.12^Aa^ | 10.76±0.04^Aa^ | 11.25±0.02^Bb^ | 11.93±0.36^Aa^ | 10.84±0.38^Aa^ | 11.48±0.05^Aa^ |
| Phenols | 10.30±0.13^Bb^ | 10.02±0.08^Cc^ | 9.57±0.06^Bb^ | 11.46±0.13^Aa^ | 12.01±0.10^Aa^ | 11.04±0.12^Aa^ | 10.58±0.13^Bb^ | 11.38±0.25^Bb^ | 11.24±0.05^Aa^ |
| Lignans, neolignans and related compounds | 8.24±0.12^Cc^ | 7.80±0.07^Cc^ | 7.39±0.10^Cc^ | 10.31±0.18^Bb^ | 9.93±0.04^Bb^ | 9.44±0.08^Bb^ | 11.18±0.24^Aa^ | 11.30±0.27^Aa^ | 10.80±0.11^Aa^ |
| Nucleosides, nucleotides, and analogues | 10.15±0.10^AaB^ | 10.13±0.03^Aa^ | 10.21±0.05^Bb^ | 10.40±0.19^Aa^ | 10.33±0.13^Aa^ | 10.58±0.10^Aa^ | 9.59±0.18^Bb^ | 10.35±0.71^Aa^ | 10.44±0.10^AaB^ |
| Alkaloids and derivatives | 8.86±0.05^Bb^ | 8.92±0.03^Bb^ | 8.46±0.15^Bc^ | 11.28±0.13^Aa^ | 10.40±0.07^Aa^ | 9.94±0.10^Ab^ | 9.93±0.83^ABb^ | 10.39±0.08^Aa^ | 10.29±0.08^Aa^ |
| Organic nitrogen compounds | 10.46±0.09^Bb^ | 9.97±0.09^Bb^ | 10.33±0.04^Cc^ | 12.13±0.18^Aa^ | 12.25±0.07^Aa^ | 11.32±0.08^Bb^ | 12.93±0.65^Aa^ | 12.22±0.19^Aa^ | 13.28±0.27^Aa^ |

Notes: different capital letters in each column indicate significance *P* < 0.01, and different lowercase letters indicate *P* < 0.05. Data are expressed as the mean ± standard deviation (n = 3).

**Table S5.** List of unique metabolites in the three developmental stages of *Gastrodia elata.*

| **Cultivar** | **ID** | **m/z** | **Metabolites** | **Class** | **Formula** |
| --- | --- | --- | --- | --- | --- |
| *G. elata* f. *elata* | **4.92_407.1918m/z** | **407.191773667383** | **ascr#4** | **Carbohydrates and carbohydrate conjugates** | **C18H32O10** |
|  | **8.48_294.0768m/z** | **294.076830298082** | **Avenanthramide E** | **Cinnamaldehydes, Cinnamic acids and derivatives** | **C17H15NO5** |
|  | **5.45_693.2027m/z** | **693.202672316158** | **Embigenin 2''-(2'''-acetylrhamnoside)** | **Flavonoids and derivatives** | **C31H36O15** |
|  | **8.29_515.1701m/z** | **515.170118711126** | **2-Methoxyestrone 3-glucuronide** | **Lipids and lipid-like molecules** | **C25H32O9** |
|  | **8.94_275.1278m/z** | **275.127752091921** | **4,4'-dihydroxy-3,5-dimethoxydihydrostilbene** | **Stilbenes** | **C16H18O4** |
|  | **4.85_846.3174m/z** | **846.317352424982** | **beta-Isorhodomycinone** | **Others** | **C40H51NO16** |
|  | **2.19_248.1646m/z** | **248.164562872957** | **Naxagolide** | **Benzenoids and derivatives** | **C15H21NO2** |
|  | **8.61_418.1649m/z** | **418.164897158878** | **latrunculin B** | **Others** | **C20H29NO5S** |
|  | **6.93_280.0974m/z** | **280.097440565068** | **(-)-Norushinsunine** | **Alkaloids and derivatives** | **C17H15NO3** |
|  | **7.83_284.1500m/z** | **284.15000259944** | **O-Pimeloylcarnitine** | **Organic acids and derivatives** | **C14H25NO6** |
|  | **4.99_593.2613m/z** | **593.26130275473** | **Dipiperamide E** | **Organoheterocyclic compounds** | **C34H38N2O6** |
|  | **9.70_265.1547m/z** | **265.154686291563** | **Vorinostat** | **Benzenoids and derivatives** | **C14H20N2O3** |
|  | **5.45_663.1924m/z** | **663.192407604474** | **6-Hydroxyluteolin 5,6,3',4'-tetramethyl ether 7-cellobioside** | **Flavonoids and derivatives** | **C31H38O17** |
|  | **4.29_260.1134m/z** | **260.113439979586** | **6-hydroxyoct-5-enoylglycine** | **Amino acids, peptides, and analogues** | **C10H17NO4** |
|  | **7.82_388.1542m/z** | **388.154193612506** | **MS 3** | **Others** | **C21H24O7** |
|  | **4.22_280.1551m/z** | **280.155087766017** | **Feruloylcholine** | **Cinnamaldehydes, Cinnamic acids and derivatives** | **C15H22NO4+** |
|  | **6.93_174.0549m/z** | **174.054905230204** | **3-Acetoxyindole** | **Organoheterocyclic compounds** | **C10H9NO2** |
|  | **4.31_571.2024m/z** | **571.202356213844** | **16,17-Dihydro-16alpha,17-dihydroxygibberellin A7 17-glucoside** | **Lipids and lipid-like molecules** | **C25H34O12** |
|  | **5.52_430.2012m/z** | **430.201178392118** | **Fluocinolone** | **Lipids and lipid-like molecules** | **C21H26F2O6** |
|  | **4.69_656.2701m/z** | **656.270057212299** | **Kanokoside C** | **Carbohydrates and carbohydrate conjugates** | **C27H42O17** |
|  | **5.64_282.1124m/z** | **282.112411496724** | **3-carboxy-4-methyl-5-(4-oxopentyl)-2-furanpropanoic acid** | **Lipids and lipid-like molecules** | **C14H18O6** |
|  | **10.57_865.4516m/z** | **865.45163680473** | **Deltonin** | **Lipids and lipid-like molecules** | **C45H72O17** |
| *G. elata* f. *glauca* | **11.22_358.2020m/z** | **358.201978553701** | **Norethindrone oxime** | **Lipids and lipid-like molecules** | **C20H27NO2** |
|  | **1.66_402.1985m/z** | **402.198485410625** | **2,3-Butanediol apiosylglucoside** | **Carbohydrates and carbohydrate conjugates** | **C15H28O11** |
|  | **9.02_455.1489m/z** | **455.148857907641** | **Pelargonidin 3-O-glucoside** | **Stilbenes** | **C28H22O6** |
|  | **5.30_332.2067m/z** | **332.20674583838** | **(-)-trans-Carveol glucoside** | **Lipids and lipid-like molecules** | **C16H26O6** |
|  | **7.76_321.0764m/z** | **321.076402098102** | **Ambanol** | **Flavonoids and derivatives** | **C19H16O6** |
|  | **10.44_214.2166m/z** | **214.216646614091** | **2-Methylcyclododecanone** | **Organic oxygen compounds** | **C13H24O** |
|  | **9.02_622.1710m/z** | **622.171020770153** | **Relugolix** | **Benzenoids and derivatives** | **C13H24O** |
|  | **6.16_365.1496m/z** | **365.149601951212** | **Penicillin K** | **Amino acids, peptides, and analogues** | **C16H26N2O4S** |
|  | **1.63_194.0814m/z** | **194.081420068796** | **alpha-Methyl-m-tyrosine** | **Others** | **C10H13NO3** |
|  | **8.22_739.3748m/z** | **739.374841841992** | **Capsoside A** | **Lipids and lipid-like molecules** | **C33H58O15** |
|  | **8.02_206.1541m/z** | **206.154067311535** | **Diethylpropion** | **Organic oxygen compounds** | **C13H19NO** |
|  | **9.53_201.1487m/z** | **201.148721457314** | **omega-hydroxy hendecanoic acid** | **Organic acids and derivatives** | **C11H22O3** |
|  | **5.30_287.1489m/z** | **287.148874528952** | **Tensyuic acid E** | **Organic acids and derivatives** | **C14H22O6** |
|  | **4.36_195.0653m/z** | **195.065346291932** | **5-Hydroxyconiferyl alcohol** | **Phenols** | **C10H12O4** |
|  | **10.31_351.1000m/z** | **351.100019322821** | **3-[3-Fluoro-4-[6-(2-methyl-2H-tetrazol-5-YL)-3-pyridinyl] phenyl]-5-(hydroxymethyl)-2-oxazolidinone** | **Organoheterocyclic compounds** | **C17H15FN6O3** |
|  | **11.01_240.2321m/z** | **240.232132883429** | **Rosifoliol** | **Lipids and lipid-like molecules** | **C15H26O** |
|  | **9.28_198.1854m/z** | **198.185378219792** | **3,6,8-dodecatrien-1-ol** | **Lipids and lipid-like molecules** | **C12H20O** |
|  | **4.59_366.1704m/z** | **366.17039367978** | **(S)-Codamine** | **Organoheterocyclic compounds** | **C20H25NO4** |
| dark red *G. elata* | **7.68_443.2282m/z** | **443.228205060407** | **xi-Linalool 3-[rhamnosyl-(1->6)-glucoside]** | **Lipids and lipid-like molecules** | **C22H38O10** |
|  | **10.05_241.1804m/z** | **241.180357946772** | **L-Menthyl (R, S)-3-hydroxybutyrate** | **Lipids and lipid-like molecules** | **C14H26O3** |
|  | **4.94_352.1035m/z** |  | **Sambucus nigra Degraded cyanogenic glycosides (2'-Epimer)** | **Carbohydrates and carbohydrate conjugates** | **C16H19NO8** |

**Table S6. Differential metabolite pathway enrichment information.**

|  | **ID** | **Pathway** | **p-value** | **Metabolites (KEGG number)** | **Number of metabolites enriched** |
| --- | --- | --- | --- | --- | --- |
| **T-a-H VS T-b-H** | **msin00941** | **Flavonoid biosynthesis** | **0.002652** | **C00974, C01617, C05907, C05908, C09762, C09826, C12136** | **7** |
|  | **msin00592** | **alpha-Linolenic acid metabolism** | **0.005005** | **C04780, C11512, C16316, C16320, C16326** | **5** |
|  | **msin00942** | **Anthocyanin biosynthesis** | **0.026459** | **C05908, C08725, C12137, C12642, C16368** | **5** |
|  | **msin00270** | **Cysteine and methionine metabolism** | **0.028027** | **C00019, C00051, C00170, C00263, C03089** | **5** |
|  | **msin00591** | **Linoleic acid metabolism** | **0.037294** | **C14762, C14828, C14833** | **3** |
|  | **msin00908** | **Zeatin biosynthesis** | **0.077732** | **C00015, C00019, C00170** | **3** |
|  | **msin00290** | **Valine, leucine and isoleucine biosynthesis** | **0.117195** | **C00183, C00407** | **2** |
|  | **msin00965** | **Betalain biosynthesis** | **0.117195** | **C00355, C08552** | **2** |
|  | **msin00230** | **Purine metabolism** | **0.117405** | **C00037, C00301, C01367, C03838, C04376** | **5** |
|  | **msin00260** | **Glycine, serine and threonine metabolism** | **0.125114** | **C00037, C00263, C00740** | **3** |
|  | **msin00565** | **Ether lipid metabolism** | **0.134531** | **C03372, C15646** | **2** |
|  | **msin00944** | **Flavone and flavonol biosynthesis** | **0.142790** | **C01750, C04858, C05625** | **3** |
|  | **msin00970** | **Aminoacyl-tRNA biosynthesis** | **0.148861** | **C00037, C00183, C00407** | **3** |
|  | **msin00440** | **Phosphonate and phosphinate metabolism** | **0.155014** | **C00037, C03167, C05672** | **3** |
|  | **msin00940** | **Phenylpropanoid biosynthesis** | **0.186901** | **C01175, C01494, C05610** | **3** |
|  | **msin00730** | **Thiamine metabolism** | **0.189354** | **C00037, C00378** | **2** |
|  | **msin00300** | **Lysine biosynthesis** | **0.227374** | **C00263, C00449** | **2** |
|  | **msin00030** | **Pentose phosphate pathway** | **0.246603** | **C00085, C06473** | **2** |
|  | **msin00500** | **Starch and sucrose metabolism** | **0.246603** | **C00085, C00501** | **2** |
|  | **msin00950** | **Isoquinoline alkaloid biosynthesis** | **0.247312** | **C00355, C02105, C05191, C09593, C16700** | **5** |
|  | **msin00480** | **Glutathione metabolism** | **0.256244** | **C00037, C00051** | **2** |
|  | **msin00785** | **Lipoic acid metabolism** | **0.275544** | **C00037, C06423** | **2** |
|  | **msin00280** | **Valine, leucine and isoleucine degradation** | **0.285190** | **C00183, C00407** | **2** |
|  | **msin00590** | **Arachidonic acid metabolism** | **0.332917** | **C03577, C04853, C14778** | **3** |
|  | **msin00051** | **Fructose and mannose metabolism** | **0.416826** | **C00085, C02431** | **2** |
|  | **msin00760** | **Nicotinate and nicotinamide metabolism** | **0.416826** | **C00455, C19567** | **2** |
|  | **msin00710** | **Carbon fixation in photosynthetic organisms** | **0.422787** | **C00085** | **1** |
|  | **msin00310** | **Lysine degradation** | **0.425822** | **C00037, C00449** | **2** |
|  | **msin00053** | **Ascorbate and aldarate metabolism** | **0.434747** | **C00818, C03033** | **2** |
|  | **msin00790** | **Folate biosynthesis** | **0.443598** | **C00568, C04895** | **2** |
|  | **msin00945** | **Stilbenoid, diarylheptanoid and gingerol biosynthesis** | **0.480340** | **C17742** | **1** |
|  | **msin00250** | **Alanine, aspartate and glutamate metabolism** | **0.519743** | **C00402** | **1** |
|  | **msin00330** | **Arginine and proline metabolism** | **0.551211** | **C00019, C02305** | **2** |
|  | **msin00010** | **Glycolysis / Gluconeogenesis** | **0.556188** | **C00085** | **1** |
|  | **msin00960** | **Tropane, piperidine and pyridine alkaloid biosynthesis** | **0.558873** | **C00407, C01479** | **2** |
|  | **msin00920** | **Sulfur metabolism** | **0.589893** | **C00263** | **1** |
|  | **msin00966** | **Glucosinolate biosynthesis** | **0.595795** | **C00183, C00407** | **2** |
|  | **msin00350** | **Tyrosine metabolism** | **0.602901** | **C00355, C03077** | **2** |
|  | **msin00380** | **Tryptophan metabolism** | **0.637034** | **C00632, C05660** | **2** |
|  | **msin00261** | **Monobactam biosynthesis** | **0.640530** | **C00019** | **1** |
|  | **msin00360** | **Phenylalanine metabolism** | **0.723971** | **C12621** | **1** |
|  | **msin00860** | **Porphyrin metabolism** | **0.725587** | **C00037, C05768, C05775** | **3** |
|  | **msin00061** | **Fatty acid biosynthesis** | **0.770666** | **C06423** | **1** |
|  | **msin00040** | **Pentose and glucuronate interconversions** | **0.788200** | **C03033** | **1** |
|  | **msin00630** | **Glyoxylate and dicarboxylate metabolism** | **0.814522** | **C00037** | **1** |
|  | **msin00999** | **Biosynthesis of various plant secondary metabolites** | **0.903548** | **C00019, C01752** | **2** |
| **T-b-H VS S-H** | **msin00592** | **alpha-Linolenic acid metabolism** | **0.000892** | **C01226, C04780, C08491, C16316, C16320, C16321** | **6** |
|  | **msin00270** | **Cysteine and methionine metabolism** | **0.001676** | **C00019, C00051, C00097, C00197, C00263, C01005, C08276** | **7** |
|  | **msin00941** | **Flavonoid biosynthesis** | **0.043863** | **C00509, C00974, C09099, C09826, C12208** | **5** |
|  | **msin00230** | **Purine metabolism** | **0.048413** | **C00037, C00212, C00301, C00360, C03838, C05993** | **6** |
|  | **msin00908** | **Zeatin biosynthesis** | **0.081628** | **C00019, C00190, C04713** | **3** |
|  | **msin00780** | **Biotin metabolism** | **0.176347** | **C01092, C01909** | **2** |
|  | **msin00053** | **Ascorbate and aldarate metabolism** | **0.188197** | **C00072, C00191, C03033** | **3** |
|  | **msin00940** | **Phenylpropanoid biosynthesis** | **0.194925** | **C02325, C02666, C12208** | **3** |
|  | **msin00010** | **Glycolysis / Gluconeogenesis** | **0.195487** | **C00197, C06186** | **2** |
|  | **msin00730** | **Thiamine metabolism** | **0.195487** | **C00037, C00097** | **2** |
|  | **msin00410** | **beta-Alanine metabolism** | **0.205156** | **C00135, C00750** | **2** |
|  | **msin00400** | **Phenylalanine, tyrosine and tryptophan biosynthesis** | **0.234441** | **C00279, C00826** | **2** |
|  | **msin00561** | **Glycerolipid metabolism** | **0.263962** | **C00197, C05401** | **2** |
|  | **msin00261** | **Monobactam biosynthesis** | **0.273818** | **C00019, C00062** | **2** |
|  | **msin00330** | **Arginine and proline metabolism** | **0.286561** | **C00019, C00062, C00750** | **3** |
|  | **msin04075** | **Plant hormone signal transduction** | **0.293244** | **C08491** | **1** |
|  | **msin00460** | **Cyanoamino acid metabolism** | **0.332652** | **C00037, C00097** | **2** |
|  | **msin00052** | **Galactose metabolism** | **0.342354** | **C00795, C05401** | **2** |
|  | **msin00590** | **Arachidonic acid metabolism** | **0.344787** | **C03577, C04707, C14717** | **3** |
|  | **msin00999** | **Biosynthesis of various plant secondary metabolites** | **0.354635** | **C00019, C01424, C02162, C02274, C10556** | **5** |
|  | **msin00310** | **Lysine degradation** | **0.436355** | **C00037, C00739** | **2** |
|  | **msin00564** | **Glycerophospholipid metabolism** | **0.436355** | **C00588, C04230** | **2** |
|  | **msin00790** | **Folate biosynthesis** | **0.454323** | **C00568, C01304** | **2** |
|  | **msin00220** | **Arginine biosynthesis** | **0.459301** | **C00062** | **1** |
|  | **msin00040** | **Pentose and glucuronate interconversions** | **0.463187** | **C00191, C03033** | **2** |
|  | **msin00430** | **Taurine and hypotaurine metabolism** | **0.473612** | **C00097** | **1** |
|  | **msin00565** | **Ether lipid metabolism** | **0.487547** | **C15646** | **1** |
|  | **msin00630** | **Glyoxylate and dicarboxylate metabolism** | **0.506223** | **C00037, C00197** | **2** |
|  | **msin00250** | **Alanine, aspartate and glutamate metabolism** | **0.527200** | **C00402** | **1** |
|  | **msin00591** | **Linoleic acid metabolism** | **0.539731** | **C14825** | **1** |
|  | **msin00750** | **Vitamin B6 metabolism** | **0.539731** | **C00279** | **1** |
|  | **msin00620** | **Pyruvate metabolism** | **0.575383** | **C05993** | **1** |
|  | **msin00300** | **Lysine biosynthesis** | **0.608301** | **C00263** | **1** |
|  | **msin00600** | **Sphingolipid metabolism** | **0.608301** | **C00319** | **1** |
|  | **msin00785** | **Lipoic acid metabolism** | **0.657642** | **C00037** | **1** |
|  | **msin00640** | **Propanoate metabolism** | **0.666745** | **C05983** | **1** |
|  | **msin00562** | **Inositol phosphate metabolism** | **0.716546** | **C00191** | **1** |
|  | **msin00650** | **Butanoate metabolism** | **0.716546** | **C00497** | **1** |
|  | **msin00860** | **Porphyrin metabolism** | **0.738665** | **C00037, C04778, C05787** | **3** |
|  | **msin00944** | **Flavone and flavonol biosynthesis** | **0.745577** | **C12629** | **1** |
|  | **msin00440** | **Phosphonate and phosphinate metabolism** | **0.758969** | **C00037** | **1** |
|  | **msin00051** | **Fructose and mannose metabolism** | **0.771663** | **C02431** | **1** |
|  | **msin00760** | **Nicotinate and nicotinamide metabolism** | **0.771663** | **C00455** | **1** |
|  | **msin00240** | **Pyrimidine metabolism** | **0.830523** | **C00364** | **1** |
|  | **msin00960** | **Tropane, piperidine and pyridine alkaloid biosynthesis** | **0.856012** | **C01479** | **1** |
|  | **msin00904** | **Diterpenoid biosynthesis** | **0.965341** | **C02034** | **1** |
|  | **msin00950** | **Isoquinoline alkaloid biosynthesis** | **0.971433** | **C00355** | **1** |
| **T-a-W VS T-b-W** | **msin00230** | **Purine metabolism** | **0.003204** | **C00059, C00262, C00294, C00301, C00360, C00385, C01367, C01762, C03838** | **9** |
|  | **msin00270** | **Cysteine and methionine metabolism** | **0.003813** | **C00019, C00051, C00059, C00197, C00263, C01005, C04188** | **7** |
|  | **msin02010** | **ABC transporters** | **0.006692** | **C00051, C00059, C00062, C00135, C00183, C01674, C01946, C02273** | **8** |
|  | **msin04075** | **Plant hormone signal transduction** | **0.057322** | **C08491, C18699** | **2** |
|  | **msin00941** | **Flavonoid biosynthesis** | **0.072665** | **C00852, C05907, C05908, C09762, C09826** | **5** |
|  | **msin00940** | **Phenylpropanoid biosynthesis** | **0.098002** | **C00852, C01494, C05608, C12205** | **4** |
|  | **msin00908** | **Zeatin biosynthesis** | **0.113437** | **C00015, C00019, C04713** | **3** |
|  | **msin00710** | **Carbon fixation in photosynthetic organisms** | **0.132160** | **C00085, C00197** | **2** |
|  | **msin00942** | **Anthocyanin biosynthesis** | **0.139162** | **C05908, C08725, C12137, C12642** | **4** |
|  | **msin00010** | **Glycolysis / Gluconeogenesis** | **0.242200** | **C00085, C00197** | **2** |
|  | **msin00920** | **Sulfur metabolism** | **0.276368** | **C00059, C00263** | **2** |
|  | **msin00300** | **Lysine biosynthesis** | **0.287755** | **C00263, C04462** | **2** |
|  | **msin00600** | **Sphingolipid metabolism** | **0.287755** | **C00319, C12144** | **2** |
|  | **msin04122** | **Sulfur relay system** | **0.288017** | **C00019** | **1** |
|  | **msin00190** | **Oxidative phosphorylation** | **0.309704** | **C00004** | **1** |
|  | **msin00030** | **Pentose phosphate pathway** | **0.310467** | **C00085, C00197** | **2** |
|  | **msin00500** | **Starch and sucrose metabolism** | **0.310467** | **C00085, C00501** | **2** |
|  | **msin00280** | **Valine, leucine and isoleucine degradation** | **0.355393** | **C00183, C00407** | **2** |
|  | **msin00330** | **Arginine and proline metabolism** | **0.366477** | **C00019, C00062, C02305** | **3** |
|  | **msin00592** | **alpha-Linolenic acid metabolism** | **0.388423** | **C08491, C11512** | **2** |
|  | **msin00900** | **Terpenoid backbone biosynthesis** | **0.410035** | **C11436, C18321** | **2** |
|  | **msin00760** | **Nicotinate and nicotinamide metabolism** | **0.502256** | **C00253, C00455** | **2** |
|  | **msin00310** | **Lysine degradation** | **0.511931** | **C00739, C03366** | **2** |
|  | **msin00740** | **Riboflavin metabolism** | **0.524078** | **C04454** | **1** |
|  | **msin00040** | **Pentose and glucuronate interconversions** | **0.540218** | **C02273, C03033** | **2** |
|  | **msin00950** | **Isoquinoline alkaloid biosynthesis** | **0.568673** | **C00355, C05189, C05191, C16700** | **4** |
|  | **msin00250** | **Alanine, aspartate and glutamate metabolism** | **0.579677** | **C00402** | **1** |
|  | **msin00410** | **beta-Alanine metabolism** | **0.628831** | **C00135** | **1** |
|  | **msin00400** | **Phenylalanine, tyrosine and tryptophan biosynthesis** | **0.661918** | **C00826** | **1** |
|  | **msin00999** | **Biosynthesis of various plant secondary metabolites** | **0.671059** | **C00019, C01752, C01772, C02274** | **4** |
|  | **msin00966** | **Glucosinolate biosynthesis** | **0.685703** | **C00183, C00407** | **2** |
|  | **msin00480** | **Glutathione metabolism** | **0.692079** | **C00051** | **1** |
|  | **msin00561** | **Glycerolipid metabolism** | **0.692079** | **C00197** | **1** |
|  | **msin00785** | **Lipoic acid metabolism** | **0.710688** | **C06423** | **1** |
|  | **msin00650** | **Butanoate metabolism** | **0.767465** | **C00497** | **1** |
|  | **msin00360** | **Phenylalanine metabolism** | **0.781553** | **C01772** | **1** |
|  | **msin00944** | **Flavone and flavonol biosynthesis** | **0.794794** | **C05625** | **1** |
|  | **msin00440** | **Phosphonate and phosphinate metabolism** | **0.807240** | **C05672** | **1** |
|  | **msin00051** | **Fructose and mannose metabolism** | **0.818937** | **C00085** | **1** |
|  | **msin00061** | **Fatty acid biosynthesis** | **0.824519** | **C06423** | **1** |
|  | **msin00053** | **Ascorbate and aldarate metabolism** | **0.829930** | **C03033** | **1** |
|  | **msin00790** | **Folate biosynthesis** | **0.835176** | **C00568** | **1** |
|  | **msin00902** | **Monoterpenoid biosynthesis** | **0.849970** | **C09844** | **1** |
|  | **msin00630** | **Glyoxylate and dicarboxylate metabolism** | **0.863448** | **C00197** | **1** |
|  | **msin00590** | **Arachidonic acid metabolism** | **0.914809** | **C04853** | **1** |
|  | **msin00380** | **Tryptophan metabolism** | **0.924906** | **C05660** | **1** |
|  | **msin00909** | **Sesquiterpenoid and triterpenoid biosynthesis** | **0.935874** | **C06080** | **1** |
|  | **msin00904** | **Diterpenoid biosynthesis** | **0.979562** | **C11864** | **1** |
|  | **msin00860** | **Porphyrin metabolism** | **0.989227** | **C05768** | **1** |
| **T-b-W VS S-W** | **msin00941** | **Flavonoid biosynthesis** | **0.005167** | **C00509, C01477, C01617, C01709, C05907, C09099, C09826** | **7** |
|  | **msin00270** | **Cysteine and methionine metabolism** | **0.012313** | **C00097, C00170, C00197, C04188, C08276, C11499** | **6** |
|  | **msin00965** | **Betalain biosynthesis** | **0.027576** | **C00082, C00355, C08552** | **3** |
|  | **msin04075** | **Plant hormone signal transduction** | **0.052816** | **C08491, C18699** | **2** |
|  | **msin00944** | **Flavone and flavonol biosynthesis** | **0.059055** | **C08491, C18699** | **2** |
|  | **msin00940** | **Phenylpropanoid biosynthesis** | **0.086180** | **C00082, C02666, C05610, C12205** | **4** |
|  | **msin00908** | **Zeatin biosynthesis** | **0.102349** | **C00170, C00190, C04713** | **3** |
|  | **msin00710** | **Carbon fixation in photosynthetic organisms** | **0.122606** | **C00197, C00279** | **2** |
|  | **msin00942** | **Anthocyanin biosynthesis** | **0.123323** | **C08725, C12137, C12642, C16301** | **4** |
|  | **msin00260** | **Glycine, serine and threonine metabolism** | **0.161514** | **C00097, C00197, C00740** | **3** |
|  | **msin00945** | **Stilbenoid, diarylheptanoid and gingerol biosynthesis** | **0.162821** | **C10287, C17742** | **2** |
|  | **msin00950** | **Isoquinoline alkaloid biosynthesis** | **0.178575** | **C00082, C00355, C02105, C05189, C09593, C16700** | **6** |
|  | **msin00970** | **Aminoacyl-tRNA biosynthesis** | **0.190531** | **C00062, C00082, C00097** | **3** |
|  | **msin00564** | **Glycerophospholipid metabolism** | **0.220761** | **C00588, C03372, C06771** | **3** |
|  | **msin00010** | **Glycolysis / Gluconeogenesis** | **0.226519** | **C00197, C06186** | **2** |
|  | **msin00730** | **Thiamine metabolism** | **0.226519** | **C00082, C00097** | **2** |
|  | **msin00920** | **Sulfur metabolism** | **0.259077** | **C00097, C08276** | **2** |
|  | **msin04122** | **Sulfur relay system** | **0.276975** | **C00097** | **1** |
|  | **msin00261** | **Monobactam biosynthesis** | **0.313377** | **C00062, C00082** | **2** |
|  | **msin00592** | **alpha-Linolenic acid metabolism** | **0.366830** | **C08491, C16320** | **2** |
|  | **msin00590** | **Arachidonic acid metabolism** | **0.403742** | **C04707, C14717, C14778** | **3** |
|  | **msin00020** | **Citrate cycle (TCA cycle)** | **0.445888** | **C05381** | **1** |
|  | **msin04148** | **Efferocytosis** | **0.462053** | **C00062** | **1** |
|  | **msin00220** | **Arginine biosynthesis** | **0.492992** | **C00062** | **1** |
|  | **msin00053** | **Ascorbate and aldarate metabolism** | **0.496863** | **C00072, C03033** | **2** |
|  | **msin00790** | **Folate biosynthesis** | **0.506171** | **C00568, C04895** | **2** |
|  | **msin00430** | **Taurine and hypotaurine metabolism** | **0.507795** | **C00097** | **1** |
|  | **msin00740** | **Riboflavin metabolism** | **0.507795** | **C01352** | **1** |
|  | **msin00040** | **Pentose and glucuronate interconversions** | **0.515369** | **C03033, C04349** | **2** |
|  | **msin00565** | **Ether lipid metabolism** | **0.522168** | **C03372** | **1** |
|  | **msin00250** | **Alanine, aspartate and glutamate metabolism** | **0.562842** | **C00402** | **1** |
|  | **msin00750** | **Vitamin B6 metabolism** | **0.575622** | **C00279** | **1** |
|  | **msin00780** | **Biotin metabolism** | **0.575622** | **C01909** | **1** |
|  | **msin00620** | **Pyruvate metabolism** | **0.611784** | **C01251** | **1** |
|  | **msin00999** | **Biosynthesis of various plant secondary metabolites** | **0.635272** | **C00082, C00254, C01424, C09263** | **4** |
|  | **msin00300** | **Lysine biosynthesis** | **0.644890** | **C01251** | **1** |
|  | **msin00500** | **Starch and sucrose metabolism** | **0.665392** | **C00501** | **1** |
|  | **msin00520** | **Amino sugar and nucleotide sugar metabolism** | **0.673165** | **C00190, C00501, C04501** | **3** |
|  | **msin00561** | **Glycerolipid metabolism** | **0.675198** | **C00197** | **1** |
|  | **msin00785** | **Lipoic acid metabolism** | **0.693965** | **C05381** | **1** |
|  | **msin00640** | **Propanoate metabolism** | **0.702941** | **C05983** | **1** |
|  | **msin00900** | **Terpenoid backbone biosynthesis** | **0.744056** | **C18321** | **1** |
|  | **msin00650** | **Butanoate metabolism** | **0.751576** | **C00497** | **1** |
|  | **msin02010** | **ABC transporters** | **0.759435** | **C00062, C05349** | **2** |
|  | **msin00360** | **Phenylalanine metabolism** | **0.765964** | **C00082** | **1** |
|  | **msin00051** | **Fructose and mannose metabolism** | **0.804359** | **C02431** | **1** |
|  | **msin00760** | **Nicotinate and nicotinamide metabolism** | **0.804359** | **C00253** | **1** |
|  | **msin00902** | **Monoterpenoid biosynthesis** | **0.836504** | **C09844** | **1** |
|  | **msin00630** | **Glyoxylate and dicarboxylate metabolism** | **0.850555** | **C00197** | **1** |
|  | **msin00240** | **Pyrimidine metabolism** | **0.859251** | **C00337** | **1** |
|  | **msin00130** | **Ubiquinone and other terpenoid-quinone biosynthesis** | **0.878858** | **C00082** | **1** |
|  | **msin00330** | **Arginine and proline metabolism** | **0.878858** | **C00062** | **1** |
|  | **msin00904** | **Diterpenoid biosynthesis** | **0.882084** | **C02034, C11864** | **2** |
|  | **msin00966** | **Glucosinolate biosynthesis** | **0.898844** | **C00082** | **1** |
|  | **msin00909** | **Sesquiterpenoid and triterpenoid biosynthesis** | **0.927374** | **C06080** | **1** |
|  | **msin00860** | **Porphyrin metabolism** | **0.986771** | **C05787** | **1** |
| **T-a-XH VS T-b-XH** | **msin00230** | **Purine metabolism** | **0.001436** | **C00059, C00262, C00294, C00301, C00360, C00385, C01367, C01762, C03838, C04376** | **10** |
|  | **msin02010** | **ABC transporters** | **0.002914** | **C00051, C00059, C00062, C00135, C00183, C01674, C01946, C02273, C05349** | **9** |
|  | **msin00908** | **Zeatin biosynthesis** | **0.007966** | **C00015, C00019, C00170, C04713, C16431** | **5** |
|  | **msin00270** | **Cysteine and methionine metabolism** | **0.065376** | **C00019, C00051, C00059, C00170, C04188** | **5** |
|  | **msin00360** | **Phenylalanine metabolism** | **0.073529** | **C00166, C01772, C05853, C12621** | **4** |
|  | **msin00941** | **Flavonoid biosynthesis** | **0.091451** | **C05907, C09099, C09762, C09826, C12136** | **5** |
|  | **msin00942** | **Anthocyanin biosynthesis** | **0.166107** | **C08725, C12137, C12642, C16368** | **4** |
|  | **msin00965** | **Betalain biosynthesis** | **0.170933** | **C00355, C08552** | **2** |
|  | **msin00330** | **Arginine and proline metabolism** | **0.198904** | **C00019, C00062, C00750, C02305** | **4** |
|  | **msin00944** | **Flavone and flavonol biosynthesis** | **0.229897** | **C01750, C04858, C05625** | **3** |
|  | **msin00591** | **Linoleic acid metabolism** | **0.242990** | **C14827, C14828** | **2** |
|  | **msin00350** | **Tyrosine metabolism** | **0.247719** | **C00355, C02465, C03077, C05582** | **4** |
|  | **msin00310** | **Lysine degradation** | **0.274042** | **C00449, C00739, C03366** | **3** |
|  | **msin00410** | **beta-Alanine metabolism** | **0.279631** | **C00135, C00750** | **2** |
|  | **msin04122** | **Sulfur relay system** | **0.305368** | **C00019** | **1** |
|  | **msin00400** | **Phenylalanine, tyrosine and tryptophan biosynthesis** | **0.316161** | **C00166, C00826** | **2** |
|  | **msin00190** | **Oxidative phosphorylation** | **0.328037** | **C00004** | **1** |
|  | **msin00030** | **Pentose phosphate pathway** | **0.340300** | **C00085, C00257** | **2** |
|  | **msin00500** | **Starch and sucrose metabolism** | **0.340300** | **C00085, C00501** | **2** |
|  | **msin00480** | **Glutathione metabolism** | **0.352275** | **C00051, C00750** | **2** |
|  | **msin00999** | **Biosynthesis of various plant secondary metabolites** | **0.356249** | **C00019, C00353, C01752, C01772, C02274, C10556** | **6** |
|  | **msin00592** | **alpha-Linolenic acid metabolism** | **0.422321** | **C11512, C16326** | **2** |
|  | **msin00950** | **Isoquinoline alkaloid biosynthesis** | **0.423939** | **C00355, C02105, C05189, C09593, C16700** | **5** |
|  | **msin00460** | **Cyanoamino acid metabolism** | **0.433634** | **C00183, C00844** | **2** |
|  | **msin00710** | **Carbon fixation in photosynthetic organisms** | **0.501715** | **C00085** | **1** |
|  | **msin00440** | **Phosphonate and phosphinate metabolism** | **0.519581** | **C03167, C05672** | **2** |
|  | **msin00220** | **Arginine biosynthesis** | **0.533798** | **C00062** | **1** |
|  | **msin00290** | **Valine, leucine and isoleucine biosynthesis** | **0.533798** | **C00183** | **1** |
|  | **msin00053** | **Ascorbate and aldarate metabolism** | **0.559225** | **C00818, C03033** | **2** |
|  | **msin00565** | **Ether lipid metabolism** | **0.563831** | **C03372** | **1** |
|  | **msin00790** | **Folate biosynthesis** | **0.568770** | **C00568, C04895** | **2** |
|  | **msin00940** | **Phenylpropanoid biosynthesis** | **0.568770** | **C01494, C05608** | **2** |
|  | **msin00040** | **Pentose and glucuronate interconversions** | **0.578167** | **C02273, C03033** | **2** |
|  | **msin00073** | **Cutin, suberine and wax biosynthesis** | **0.591944** | **C19623** | **1** |
|  | **msin00250** | **Alanine, aspartate and glutamate metabolism** | **0.605320** | **C00402** | **1** |
|  | **msin00780** | **Biotin metabolism** | **0.618260** | **C05552** | **1** |
|  | **msin00010** | **Glycolysis / Gluconeogenesis** | **0.642892** | **C00085** | **1** |
|  | **msin00920** | **Sulfur metabolism** | **0.676914** | **C00059** | **1** |
|  | **msin00960** | **Tropane, piperidine and pyridine alkaloid biosynthesis** | **0.686815** | **C00166, C01479** | **2** |
|  | **msin00300** | **Lysine biosynthesis** | **0.687524** | **C00449** | **1** |
|  | **msin00600** | **Sphingolipid metabolism** | **0.687524** | **C00319** | **1** |
|  | **msin00785** | **Lipoic acid metabolism** | **0.735610** | **C06423** | **1** |
|  | **msin00280** | **Valine, leucine and isoleucine degradation** | **0.744307** | **C00183** | **1** |
|  | **msin00909** | **Sesquiterpenoid and triterpenoid biosynthesis** | **0.788129** | **C01126, C06080** | **2** |
|  | **msin00260** | **Glycine, serine and threonine metabolism** | **0.797734** | **C00740** | **1** |
|  | **msin00051** | **Fructose and mannose metabolism** | **0.840072** | **C00085** | **1** |
|  | **msin00760** | **Nicotinate and nicotinamide metabolism** | **0.840072** | **C03150** | **1** |
|  | **msin00061** | **Fatty acid biosynthesis** | **0.845354** | **C06423** | **1** |
|  | **msin00902** | **Monoterpenoid biosynthesis** | **0.869280** | **C09782** | **1** |
|  | **msin00130** | **Ubiquinone and other terpenoid-quinone biosynthesis** | **0.906666** | **C00353** | **1** |
|  | **msin00904** | **Diterpenoid biosynthesis** | **0.918049** | **C00353, C11864** | **2** |
|  | **msin00966** | **Glucosinolate biosynthesis** | **0.923782** | **C00183** | **1** |
|  | **msin00590** | **Arachidonic acid metabolism** | **0.928764** | **C04853** | **1** |
|  | **msin00906** | **Carotenoid biosynthesis** | **0.983502** | **C00353** | **1** |
|  | **msin00860** | **Porphyrin metabolism** | **0.992248** | **C05768** | **1** |
| **T-b-XH VS S-XH** | **msin00941** | **Flavonoid biosynthesis** | **0.000206** | **C00509, C01477, C01604, C01709, C05908, C09099, C09762, C09826, C12136** | **9** |
|  | **msin00591** | **Linoleic acid metabolism** | **0.001171** | **C04717, C14767, C14825, C14827, C14828** | **5** |
|  | **msin00592** | **alpha-Linolenic acid metabolism** | **0.001352** | **C01226, C04780, C11512, C16316, C16320, C16321** | **6** |
|  | **msin00230** | **Purine metabolism** | **0.002053** | **C00037, C00059, C00262, C00294, C00301, C00360, C01367, C04376, C05993** | **9** |
|  | **msin00270** | **Cysteine and methionine metabolism** | **0.002648** | **C00059, C00097, C00197, C03089, C04188, C08276, C11499** | **7** |
|  | **msin00350** | **Tyrosine metabolism** | **0.022597** | **C00082, C00355, C00822, C05579, C05596, C06046** | **6** |
|  | **msin00965** | **Betalain biosynthesis** | **0.026256** | **C00082, C00355, C08552** | **3** |
|  | **msin00942** | **Anthocyanin biosynthesis** | **0.038499** | **C05908, C08725, C12137, C16301, C16368** | **5** |
|  | **msin00710** | **Carbon fixation in photosynthetic organisms** | **0.118838** | **C00197, C00279** | **2** |
|  | **msin00260** | **Glycine, serine and threonine metabolism** | **0.155230** | **C00037, C00097, C00197** | **3** |
|  | **msin00360** | **Phenylalanine metabolism** | **0.162133** | **C00082, C00166, C05853** | **3** |
|  | **msin00944** | **Flavone and flavonol biosynthesis** | **0.176212** | **C01477, C04858, C05625** | **3** |
|  | **msin00564** | **Glycerophospholipid metabolism** | **0.212766** | **C00307, C00588, C04230** | **3** |
|  | **msin00940** | **Phenylpropanoid biosynthesis** | **0.227833** | **C00082, C02666, C05610** | **3** |
|  | **msin00620** | **Pyruvate metabolism** | **0.230873** | **C01251, C05993** | **2** |
|  | **msin00520** | **Amino sugar and nucleotide sugar metabolism** | **0.244536** | **C00190, C00501, C00935, C04501, C06241** | **5** |
|  | **msin00300** | **Lysine biosynthesis** | **0.262840** | **C01251, C04002** | **2** |
|  | **msin04122** | **Sulfur relay system** | **0.272514** | **C00097** | **1** |
|  | **msin00908** | **Zeatin biosynthesis** | **0.305485** | **C00190, C04713** | **2** |
|  | **msin00590** | **Arachidonic acid metabolism** | **0.392036** | **C04707, C05954, C14717** | **3** |
|  | **msin00380** | **Tryptophan metabolism** | **0.422998** | **C00331, C00632, C05653** | **3** |
|  | **msin00440** | **Phosphonate and phosphinate metabolism** | **0.448795** | **C00037, C05672** | **2** |
|  | **msin00232** | **Caffeine metabolism** | **0.471276** | **C07480** | **1** |
|  | **msin00220** | **Arginine biosynthesis** | **0.486419** | **C00062** | **1** |
|  | **msin00053** | **Ascorbate and aldarate metabolism** | **0.486800** | **C00072, C03033** | **2** |
|  | **msin00790** | **Folate biosynthesis** | **0.496048** | **C00272, C05922** | **2** |
|  | **msin02010** | **ABC transporters** | **0.497813** | **C00059, C00062, C05349** | **3** |
|  | **msin00430** | **Taurine and hypotaurine metabolism** | **0.501133** | **C00097** | **1** |
|  | **msin00740** | **Riboflavin metabolism** | **0.501133** | **C01352** | **1** |
|  | **msin00945** | **Stilbenoid, diarylheptanoid and gingerol biosynthesis** | **0.515429** | **C10287** | **1** |
|  | **msin00950** | **Isoquinoline alkaloid biosynthesis** | **0.516882** | **C00082, C00355, C05189, C09593** | **4** |
|  | **msin00073** | **Cutin, suberine and wax biosynthesis** | **0.542815** | **C19623** | **1** |
|  | **msin00630** | **Glyoxylate and dicarboxylate metabolism** | **0.549292** | **C00037, C00197** | **2** |
|  | **msin00240** | **Pyrimidine metabolism** | **0.566156** | **C00380, C01168** | **2** |
|  | **msin00750** | **Vitamin B6 metabolism** | **0.568667** | **C00279** | **1** |
|  | **msin00010** | **Glycolysis / Gluconeogenesis** | **0.593071** | **C00197** | **1** |
|  | **msin00960** | **Tropane, piperidine and pyridine alkaloid biosynthesis** | **0.614026** | **C00166, C01479** | **2** |
|  | **msin00600** | **Sphingolipid metabolism** | **0.637849** | **C00319** | **1** |
|  | **msin00966** | **Glucosinolate biosynthesis** | **0.650791** | **C00082, C08401** | **2** |
|  | **msin00500** | **Starch and sucrose metabolism** | **0.658371** | **C00501** | **1** |
|  | **msin00561** | **Glycerolipid metabolism** | **0.668196** | **C00197** | **1** |
|  | **msin00785** | **Lipoic acid metabolism** | **0.687014** | **C00037** | **1** |
|  | **msin00640** | **Propanoate metabolism** | **0.696023** | **C05983** | **1** |
|  | **msin00900** | **Terpenoid backbone biosynthesis** | **0.737355** | **C18321** | **1** |
|  | **msin00650** | **Butanoate metabolism** | **0.744927** | **C00497** | **1** |
|  | **msin00860** | **Porphyrin metabolism** | **0.786127** | **C00037, C04778, C05787** | **3** |
|  | **msin00051** | **Fructose and mannose metabolism** | **0.798211** | **C02431** | **1** |
|  | **msin00760** | **Nicotinate and nicotinamide metabolism** | **0.798211** | **C00455** | **1** |
|  | **msin00310** | **Lysine degradation** | **0.804043** | **C00037** | **1** |
|  | **msin00040** | **Pentose and glucuronate interconversions** | **0.820556** | **C03033** | **1** |
|  | **msin00902** | **Monoterpenoid biosynthesis** | **0.830791** | **C09782** | **1** |
|  | **msin00130** | **Ubiquinone and other terpenoid-quinone biosynthesis** | **0.873910** | **C00082** | **1** |
|  | **msin00330** | **Arginine and proline metabolism** | **0.873910** | **C00062** | **1** |
|  | **msin00909** | **Sesquiterpenoid and triterpenoid biosynthesis** | **0.923670** | **C06080** | **1** |
|  | **msin00999** | **Biosynthesis of various plant secondary metabolites** | **0.931378** | **C00082, C02162** | **2** |
|  | **msin00904** | **Diterpenoid biosynthesis** | **0.973842** | **C11864** | **1** |

1. **Supplementary Figures**

**
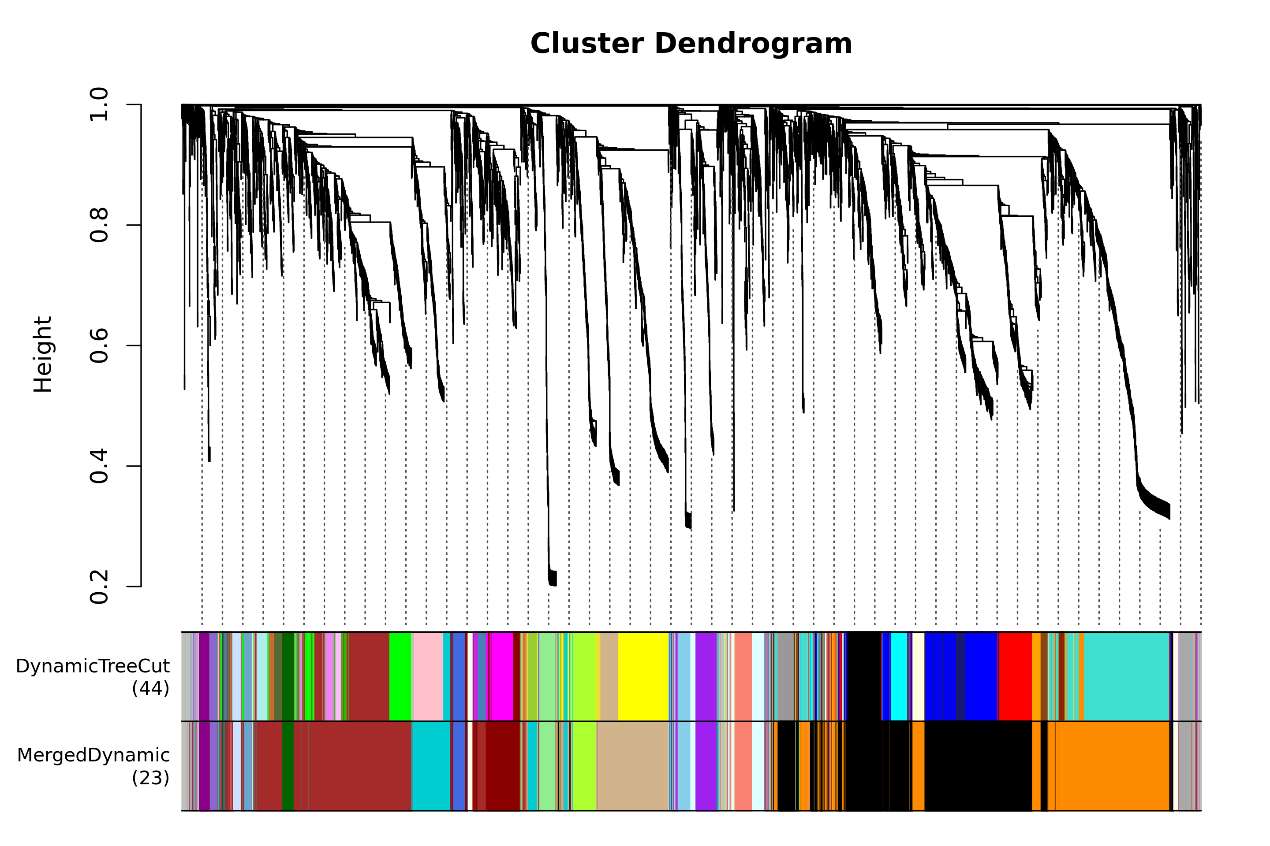
**

**Figure S1.** Module level clustering tree diagram

**
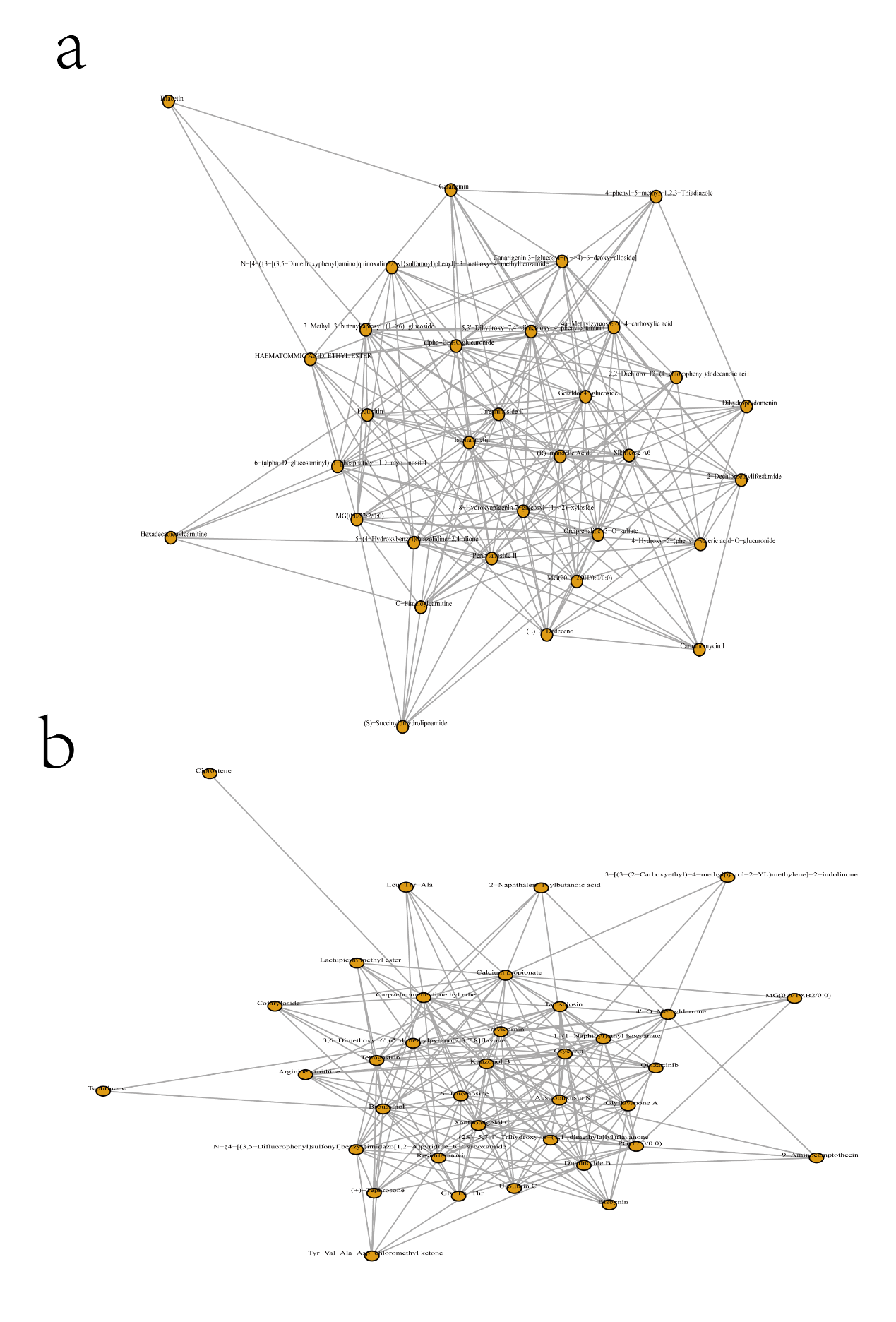
**

**Figure S2.** Network diagrams of two modules. The floralwhite module (a) and the tan module (b). The lines in the network diagrams represent the degree of connection between metabolites. The more connections a metabolite has with surrounding nodes, the more central its position in the networ

**a b**


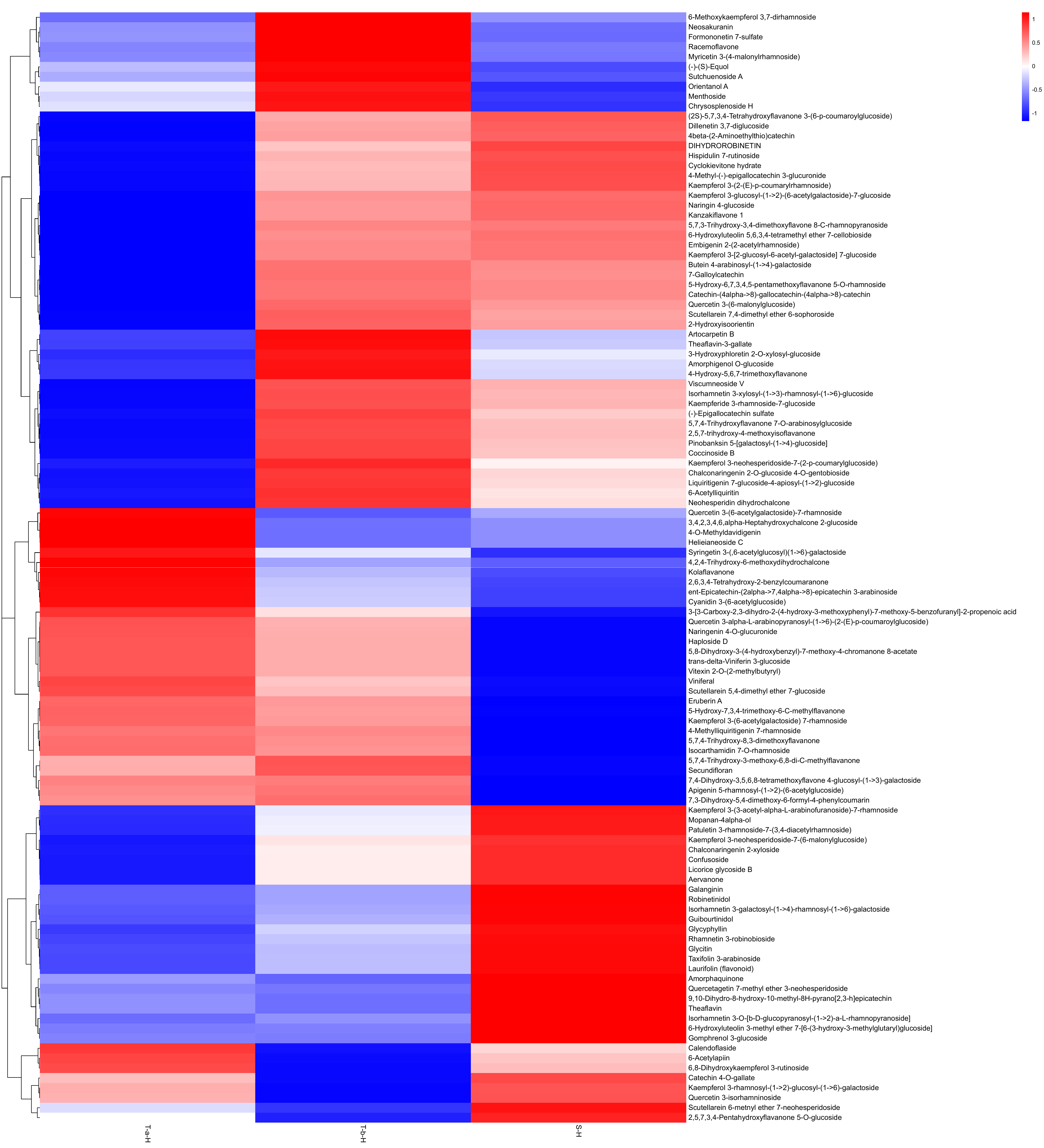

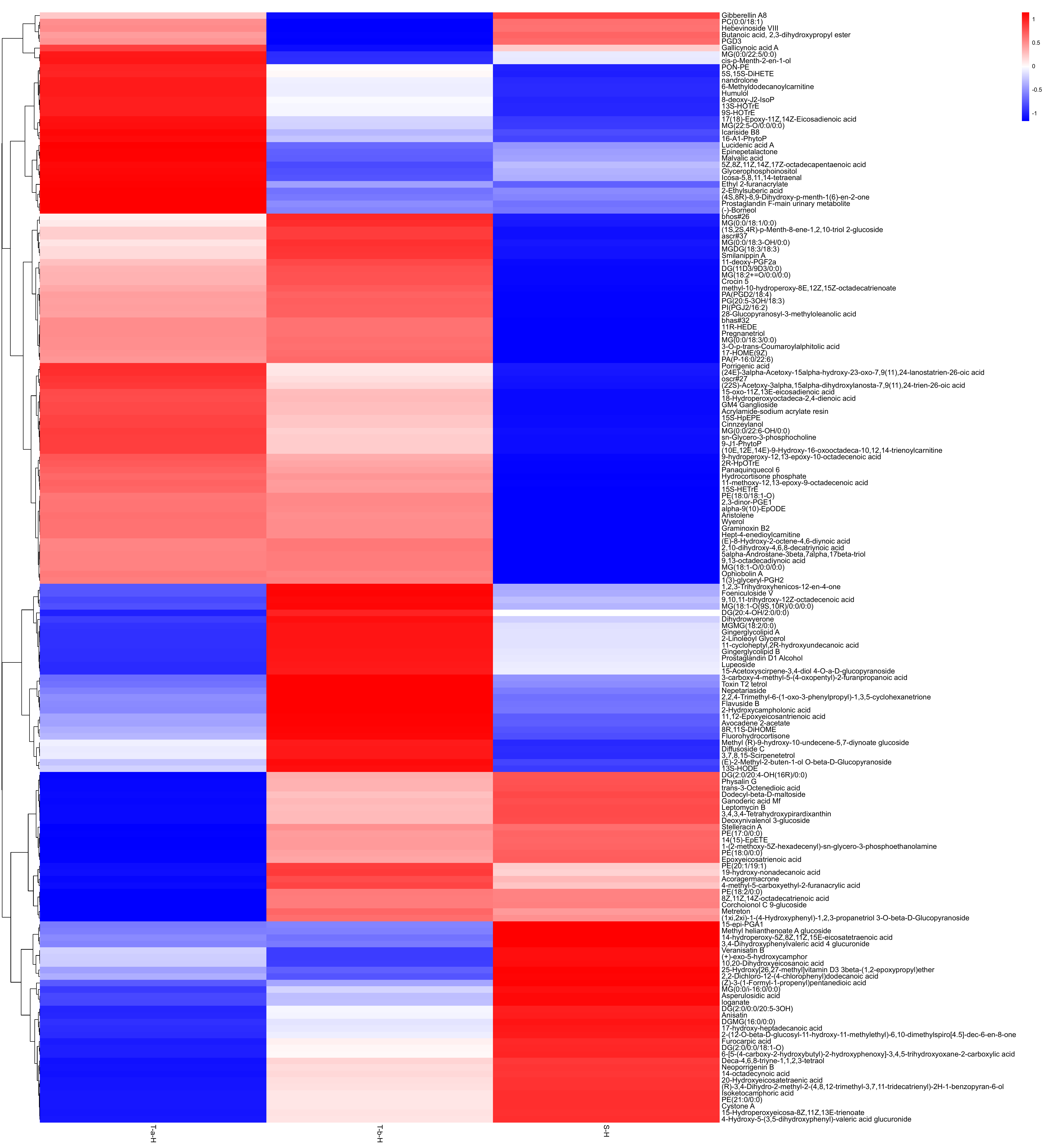


**c d**

**
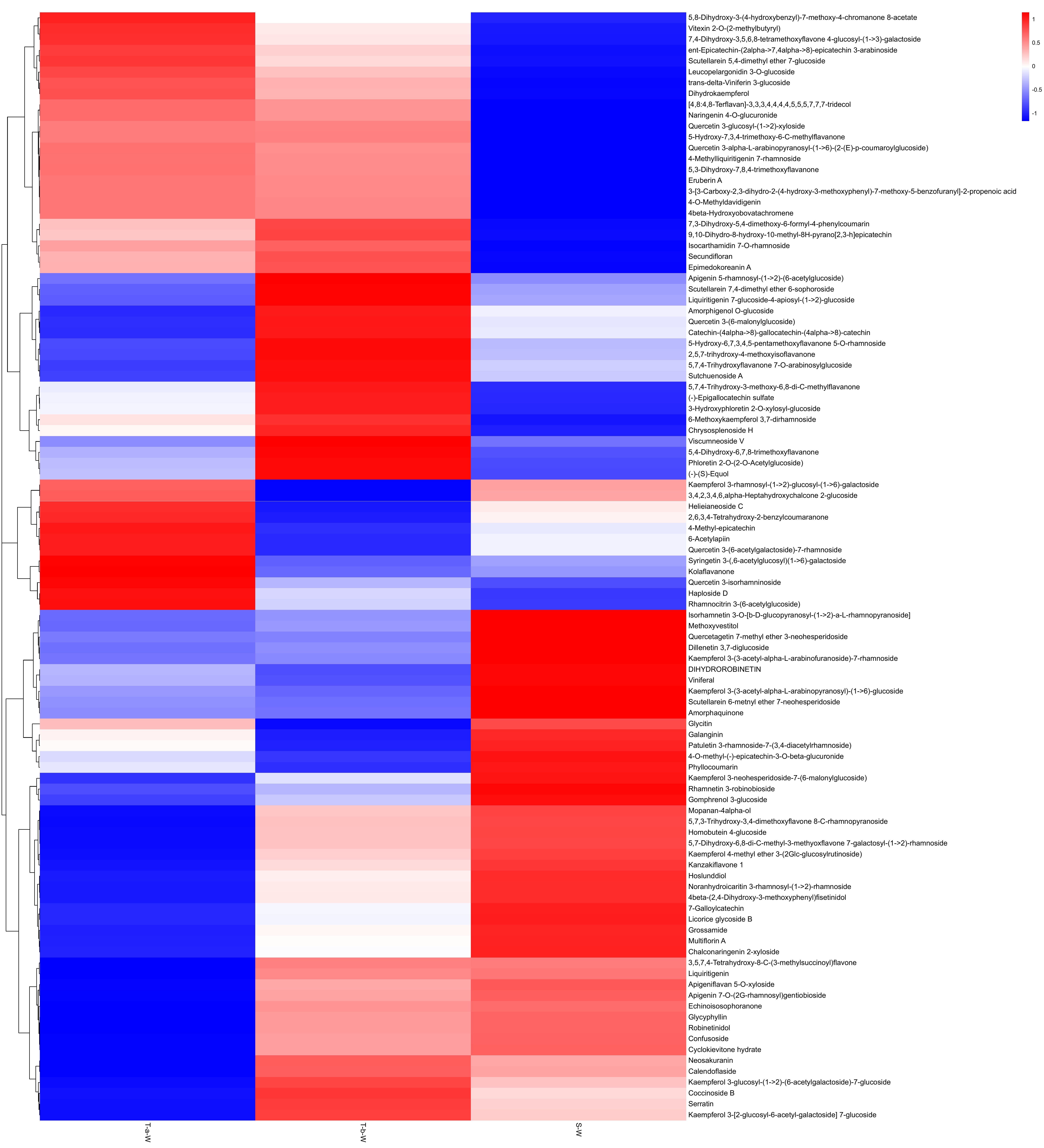

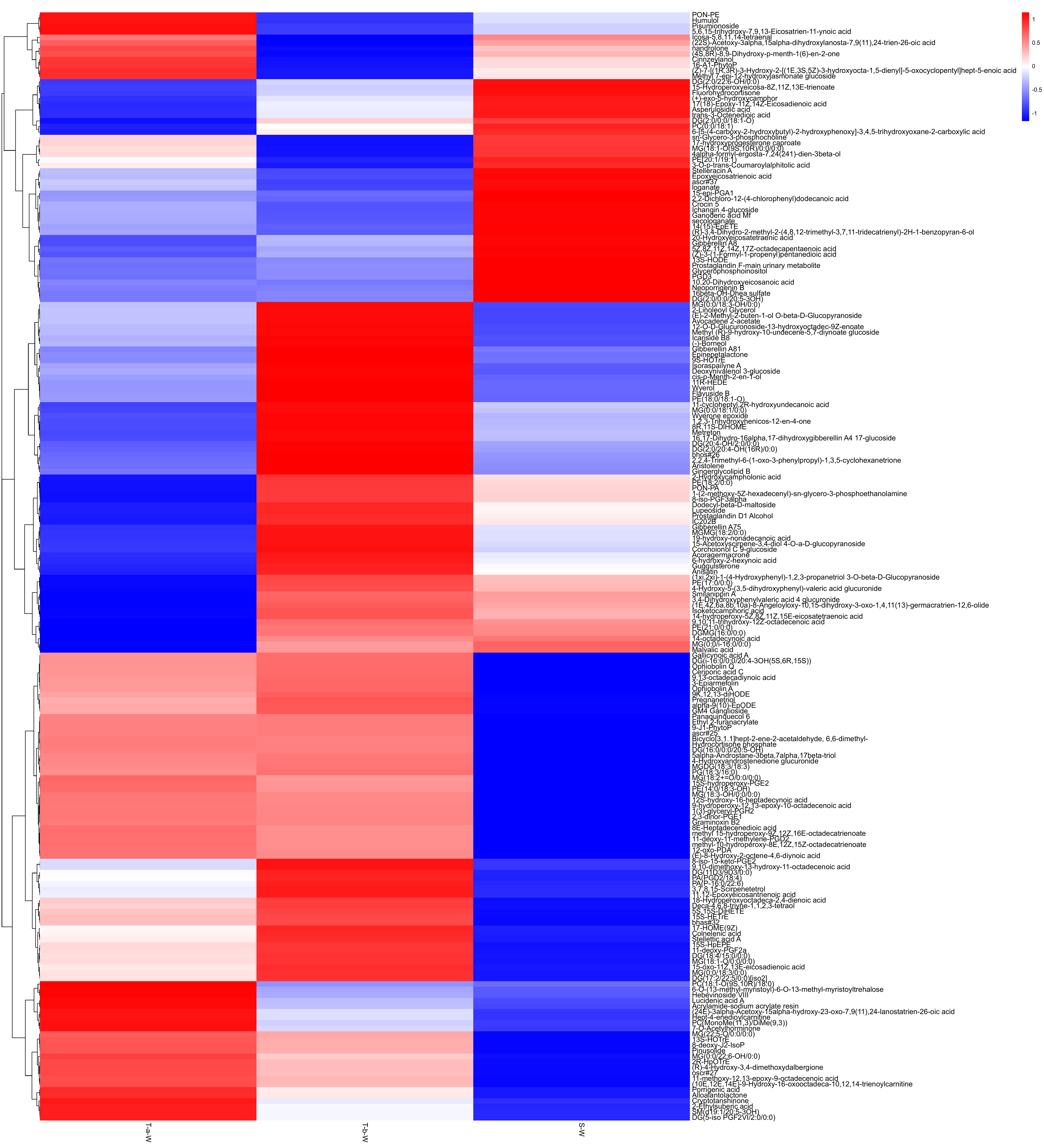
**

**e f**

**
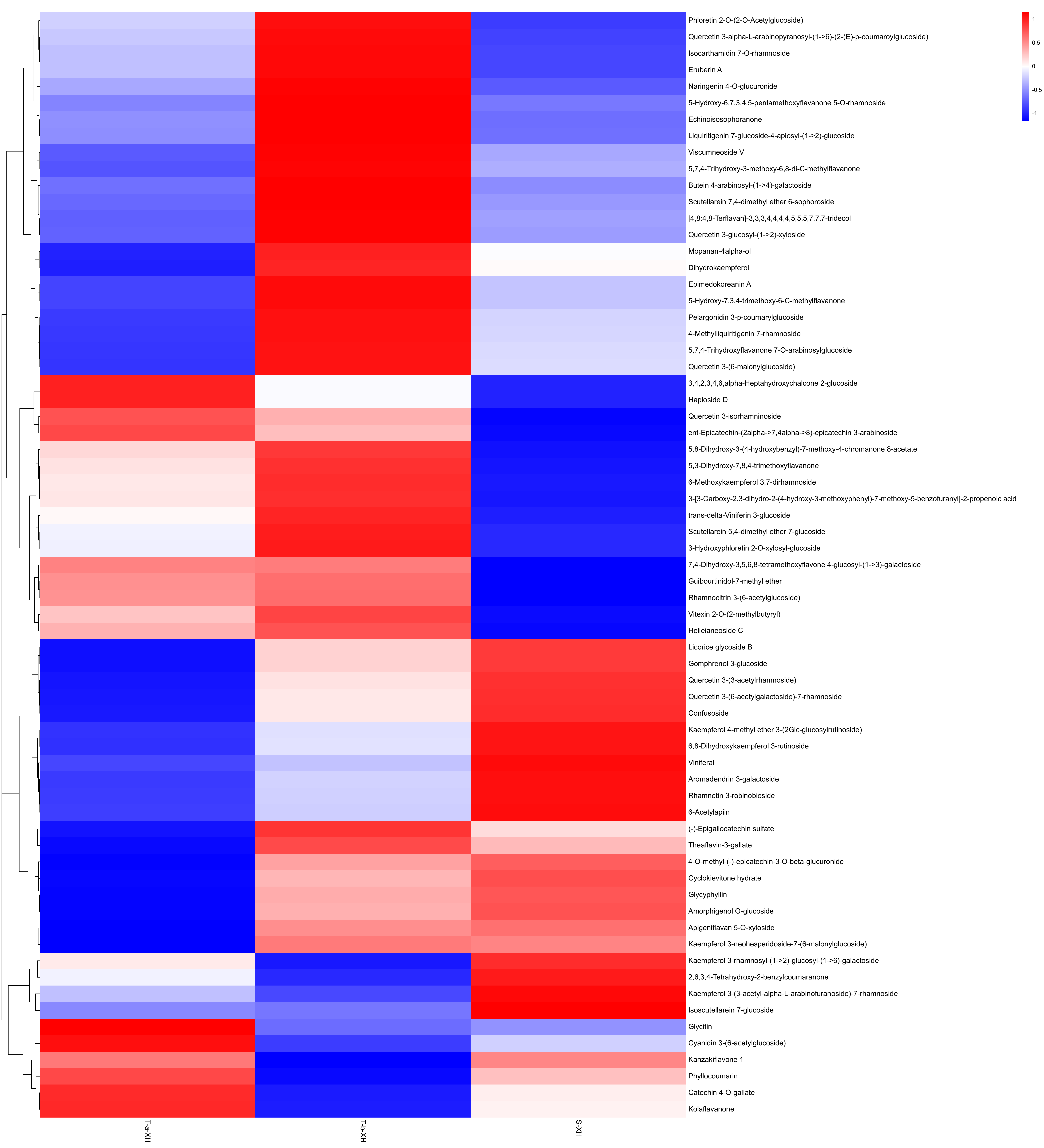

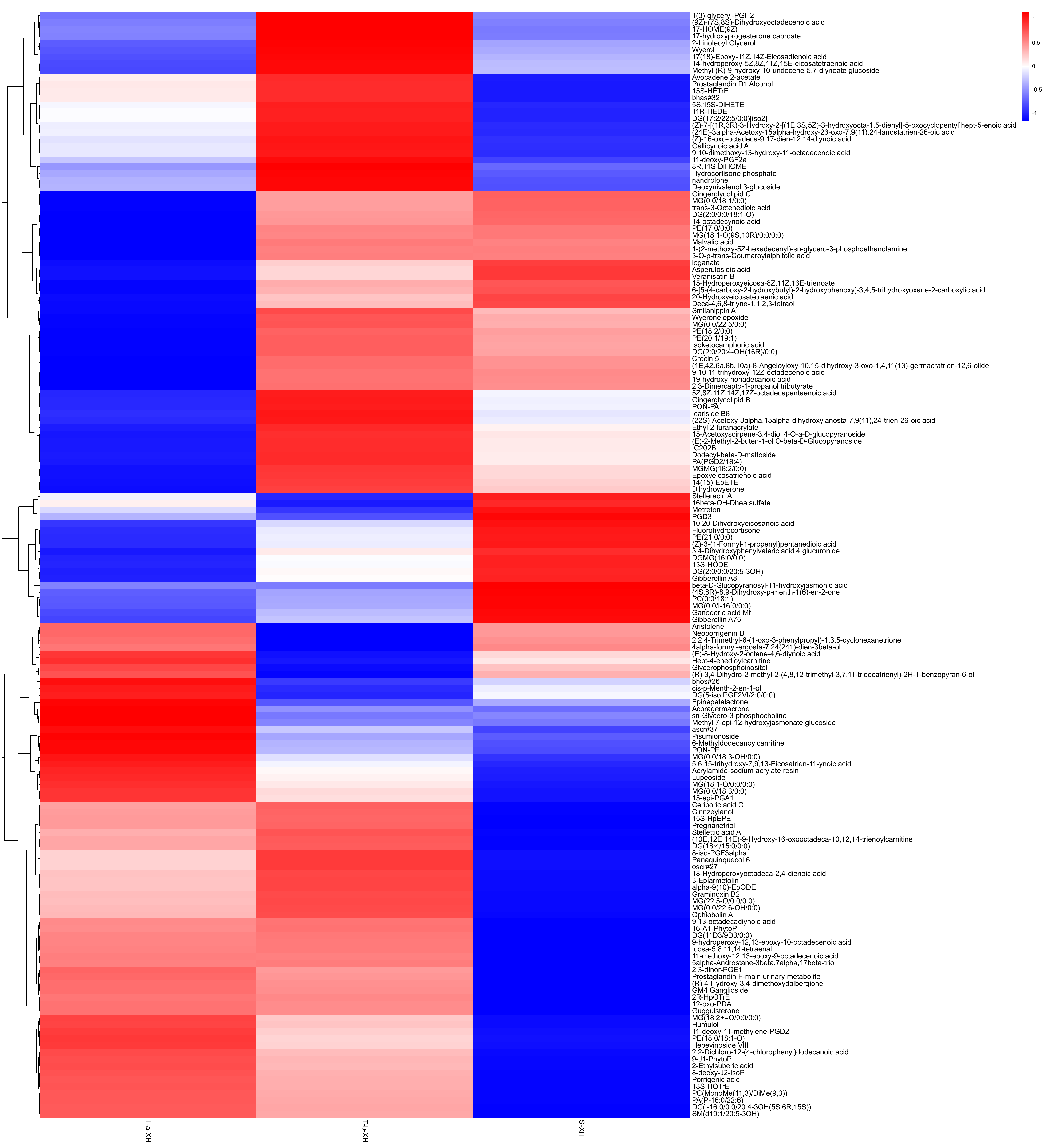
**

**Figure S3.** Cluster heatmap analysis of lipids and flavonoids at different developmental stages in three variants of *Gastrodia elata*: Lipid clustering heatmap of *G. elata* f. *elata* (a); Flavonoid clustering heatmap of *G. elata* f. *elata* (b); Lipid clustering heatmap of *G. elata* f. *glauca* (c); Flavonoid clustering heatmap of *G. elata* f. *glauca* (d); Lipid clustering heatmap of dark red *G. elata* (e); Flavonoid clustering heatmap of dark red *G. elata* (f).

**
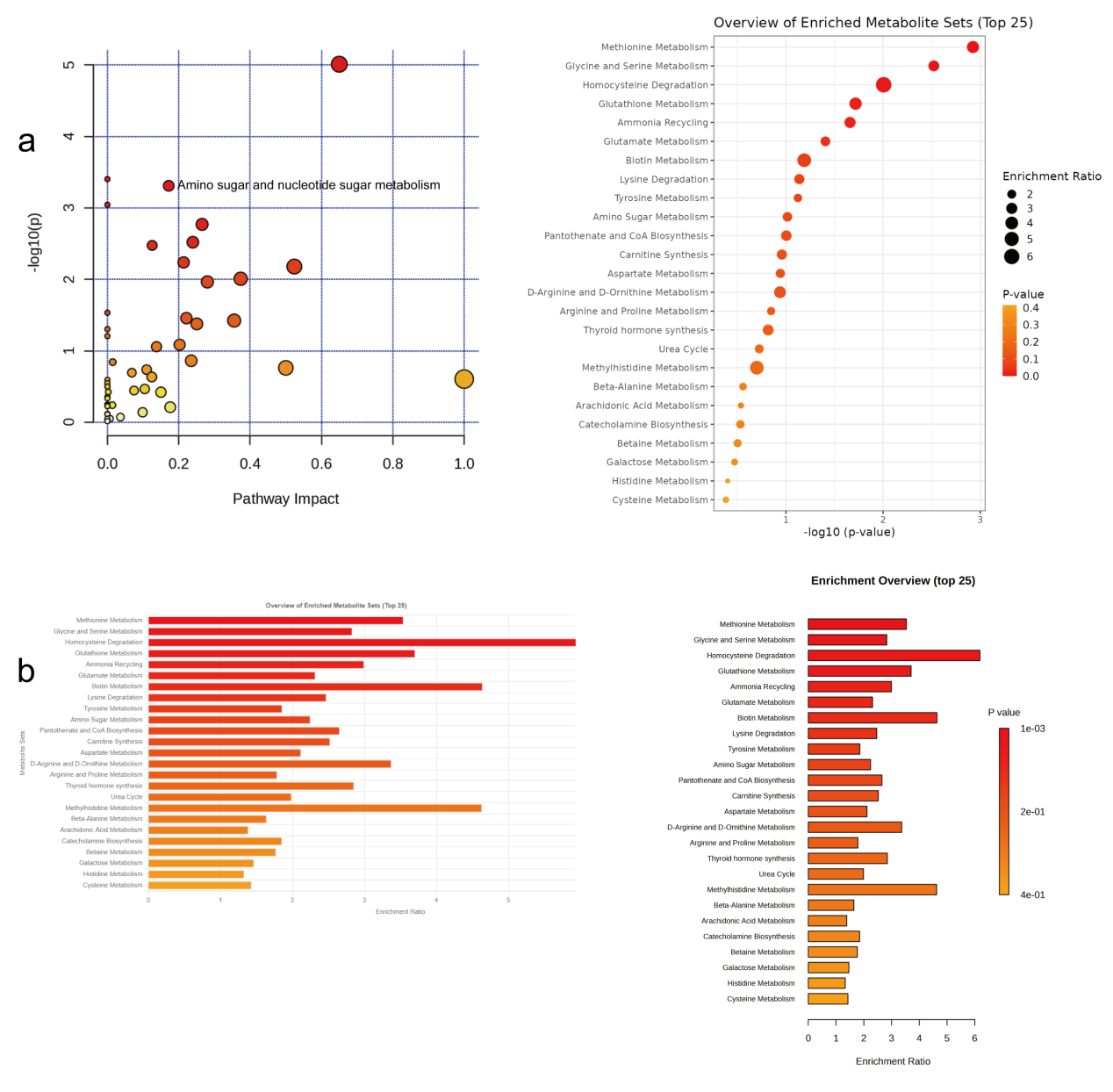
 Figure S4.** Enrichment analysis of energy metabolism pathways across the bolting stages in three varieties of *Gastrodia elata* Bubble plot displaying significantly enriched pathways. The bubble size and color intensity represent the enrichment significance or impact（a）; Bar chart of the enriched pathways. The bar length typically represents the enrichment score or the number of metabolites mapped to the pathway（b）.
